# Platelets modulate multiple markers of neutrophil function in response to *in vitro* Toll-like receptor stimulation

**DOI:** 10.1371/journal.pone.0223444

**Published:** 2019-10-03

**Authors:** Kathryn E. Hally, Georgina K. Bird, Anne C. La Flamme, Scott A. Harding, Peter D. Larsen

**Affiliations:** 1 Department of Surgery and Anaesthesia, University of Otago, Wellington, New Zealand; 2 School of Biological Sciences, Victoria University of Wellington, Wellington, New Zealand; 3 Wellington Cardiovascular Research Group, Wellington, New Zealand; 4 Department of Cardiology, Wellington Hospital, Wellington, New Zealand; University of Cambridge, UNITED KINGDOM

## Abstract

**Introduction:**

In addition to their role in facilitating leukocyte-mediated inflammation, platelets can dampen leukocyte pro-inflammatory responses in some contexts. Consequently, platelets are increasingly appreciated as regulators of inflammation. Together, platelets and neutrophils play a role in inflammation through Toll-like receptor (TLR) expression, although we do not fully understand how platelets shape neutrophil responses to TLR stimulation. Here, we aimed to determine the extent to which platelets can modulate neutrophil function in response to *in vitro* stimulation with TLR4, TLR2/1, and TLR2/6 agonists.

**Methods:**

Neutrophils from 10 healthy individuals were cultured alone or with autologous platelets. Neutrophils ± platelets were left unstimulated or were stimulated with 1 or 100 ng/mL lipopolysaccharide (LPS; a TLR4 agonist), Pam3CSK4 (a TLR2/1 agonist) and fibroblast-stimulating lipopeptide (FSL)-1 (a TLR2/6 agonist). Neutrophil activation and phagocytic activity were assessed by flow cytometry, and elastase and interleukin-8 secretion were assessed by ELISA.

**Results:**

The addition of platelets attenuated neutrophil CD66b and CD11b expression in response to various doses of Pam3CSK4 and FSL-1. Furthermore, platelet co-culture was associated with higher CD62L expression (indicating reduced CD62L shedding) in response to these TLR agonists. Platelets also reduced elastase secretion in unstimulated cultures and in response to low-dose TLR stimulation. Conversely, platelet co-culture increased neutrophil phagocytosis in unstimulated cultures and in response to low-dose Pam3CSK4 and FSL-1. Platelets also increased IL-8 secretion in response to low-dose LPS.

**Conclusion:**

Platelets are complex immunomodulators that can attenuate some, and simultaneously augment other, neutrophil functions. This modulation can occur both in the absence and presence of TLR stimulation.

## Introduction

Alongside their roles in hemostasis and thrombosis, platelets have emerged as key effectors of host-defense [1]. Platelets participate in this process principally via cross-talk with leukocytes, where platelets enhance a number of host-defense functions including neutrophil extracellular trap (NET) formation [2] and effective antigen presentation [3]. Platelets are able to mediate these inflammatory responses partly via expression of Toll-like receptors (TLRs) [4–6]. TLRs are crucial host-defense mechanisms and some TLRs are important in eliciting platelet-mediated inflammation [6–10].

The involvement of platelets in shaping inflammation is complex. Recent evidence suggests that, in addition to their role in promoting inflammation, platelets provide anti-inflammatory cues to dampen leukocyte responses during excessive inflammation [11]. For example, platelets can attenuate the production of pro-inflammatory cytokines [12, 13] and reactive oxygen species (ROS) [14, 15], and attenuate expression of activation markers [16] by immune cells in response to inflammatory stimuli. In light of these findings, platelets are increasingly appreciated as immune regulators, rather than purely pro-inflammatory cells. Interestingly, this regulatory response has been characterized in a number of inflammatory diseases where platelets are also known to be predominant drivers of leukocyte infiltration and leukocyte-mediated inflammation [17–19]. The dynamicity in platelet responses may be a mechanism to enhance, and then temper, an inflammatory response [20] to prevent rampant host damage [11] and is also both context- and stimulus-dependent [11, 21].

We have previously investigated the role of platelets in regulating leukocyte-mediated inflammation in response to TLR stimulation [16]. In addition to examining other leukocyte populations, we showed that platelets attenuated neutrophil elastase secretion and expression of the activation marker, CD66b, in response to *in vitro* stimulation with lipopolysaccharide (LPS, a TLR4 agonist), Pam3CSK4 (a TLR2/1 agonist) and fibroblast-stimulating lipopeptide (FSL)-1 (a TLR2/6 agonist). We examined this particular subset of platelet-TLRs as we have previously shown that platelets elicit very different patterns of activation in response to these prototypical TLR agonists. We [6] and others [7, 8, 22] have consistently shown that platelets become directly activated in response to Pam3CSK4, but platelets show minimal activation following incubation with either LPS or FSL-1 [2, 6, 8]. However, although not able to induce platelet activation or aggregation, LPS has been shown to facilitate platelet-neutrophil aggregation and subsequent robust production of neutrophil extracellular traps (NETs) [2]. These results suggest that platelets may mediate a more complex effect to LPS and FSL-1 via their interaction with leukocytes.

Due to the broad nature of our previous study, where we aimed to assess how platelets modulated the function of various leukocyte subsets, we did not examine neutrophil function beyond these two measurements. Thus, a detailed description of the platelet effect on neutrophil responses to TLR stimulation has yet to be conducted. This is particularly important given that platelets can exert both pro-inflammatory [2, 23, 24] and anti-inflammatory [16, 25–27] effects on neutrophils in response to various other stimuli. We suggest that different platelet phenotypes can be triggered in a stimulus-specific manner, and this has yet to be fully examined in response to TLR stimulation. Here, we aimed to determine the extent to which platelets can modulate neutrophil responses to *in vitro* stimulation with TLR4, TLR2/1, and TLR2/6 agonists.

## Materials and methods

### Neutrophil and platelet isolation

Ten healthy subjects (5 male, mean age 26 ± 3 years) were recruited into this study. Ethical approval was granted by the University of Otago Human Ethics Committee (HE16/004). Ten mL blood was drawn by venipuncture from each subject. Neutrophils were isolated from EDTA-anticoagulated blood by magnetic negative selection as per manufacturer’s instructions (Miltenyi Biotec, Bergish Gladbach, Germany). Neutrophils were then washed and resuspended in culture media (10% FCS, 2 mM L-glutamate, 100 U/mL penicillin, 100 μg/mL streptomycin, 0.01M HEPES buffer, 0.1% β-mercaptoethanol, 0.01 nM non-essential amino acids) to 10^6^ neutrophils/mL. Platelet-rich plasma (PRP) and platelet-poor plasma (PPP) were isolated from hirudin-anticoagulated blood by centrifugation at 200 x g for 12 minutes or at 1500 x g for 12 minutes, respectively. PRP was adjusted to 2.5x10^8^ platelets/mL in phosphate-buffered saline (PBS; 145 mM NaCl, 8.7 mM Na_2_HPO_4_, 1.3 mM NaH_2_PO_4_). PPP was similarly diluted in PBS. PRP and PPP were used to assess the platelet effect on markers of neutrophil activation by flow cytometry. PRP was also used to produce washed platelets (WPs). Briefly, PRP was diluted in PBS with 1 μM prostaglandin E1, pelleted, and platelets were resuspended to 2.5x10^8^ platelets/mL in culture media. WPs were used to assess the platelet effect on neutrophil phagocytosis. Platelets are quiescent during isolation from whole blood and prior to culture, as platelets did not stain positively for PAC1, a platelet activation marker, by flow cytometry (S1 Fig). Platelets also remain quiescent when cultured alone (in the absence of TLR stimulation and neutrophil co-culture) for 4 hours (also S1 Fig).

### *In vitro* TLR stimulation

To assess markers of neutrophil activation by flow cytometry, neutrophils were cultured with PRP in a ratio of 1:250 neutrophils: platelets (+ platelets). An equal amount of PPP was added to neutrophil-only cultures (- platelets). We have previously demonstrated that platelets exert their effect on leukocytes in a dose-dependent manner, and that this effect was most apparent at a neutrophil: platelet ratio of 1:250 [16]. In observance to this previous finding, we have employed the same neutrophil: platelet ratio in this study. To assess neutrophil phagocytosis, neutrophils were cultured either with WPs (+ platelets) or culture media (-platelets) and incubated with FITC-labelled rabbit IgG-coated latex beads (Cayman Chemicals, Ann Arbor, MI, USA) in a ratio of 1 μL of latex beads to every 200 μL culture media. For both neutrophil activation and phagocytosis, neutrophils ± platelets were left unstimulated or stimulated with 1 and 100 ng/mL of the following for 4 hours at 37°C/5% CO_2_: LPS from *Escherichia coli* serotype R515 (a TLR4 agonist; Enzo Life Sciences, Farmingdale, NY, USA); Pam3CSK4 (a TLR2/1 agonist; Tocris Bioscience, Bristol, UK) and FSL-1 (a TLR2/6 agonist; Santa Cruz Biotechnology, Santa Cruz, CA, USA). LPS and FSL-1 were guaranteed by the respective manufacturers to be free of any contaminants that have agonist TLR activity.

### Assessing neutrophil function

To assess neutrophil activation, neutrophils ± platelets were incubated with anti-CD16-BV421 (clone 3G8), anti-CD66b-BB515 (clone G10F5), anti-CD11b-BV510 (clone ICRF44), all sourced from Becton Dickinson, and anti-CD62L-APC (DREG-56; BioLegend, San Diego, CA, USA), or the appropriate isotype control for 50 minutes at 4°C, followed by fixation. To assess neutrophil phagocytosis, cells were incubated with Trypan blue to quench fluorescence from surface-bound latex beads-FITC as recommended by the manufacturer, and were then washed and fixed. All samples were run on a FACSCanto II flow cytometer (Becton Dickinson), and data were analyzed using FlowJo software (v10.0.7, Tree star).

**Fig 1:**
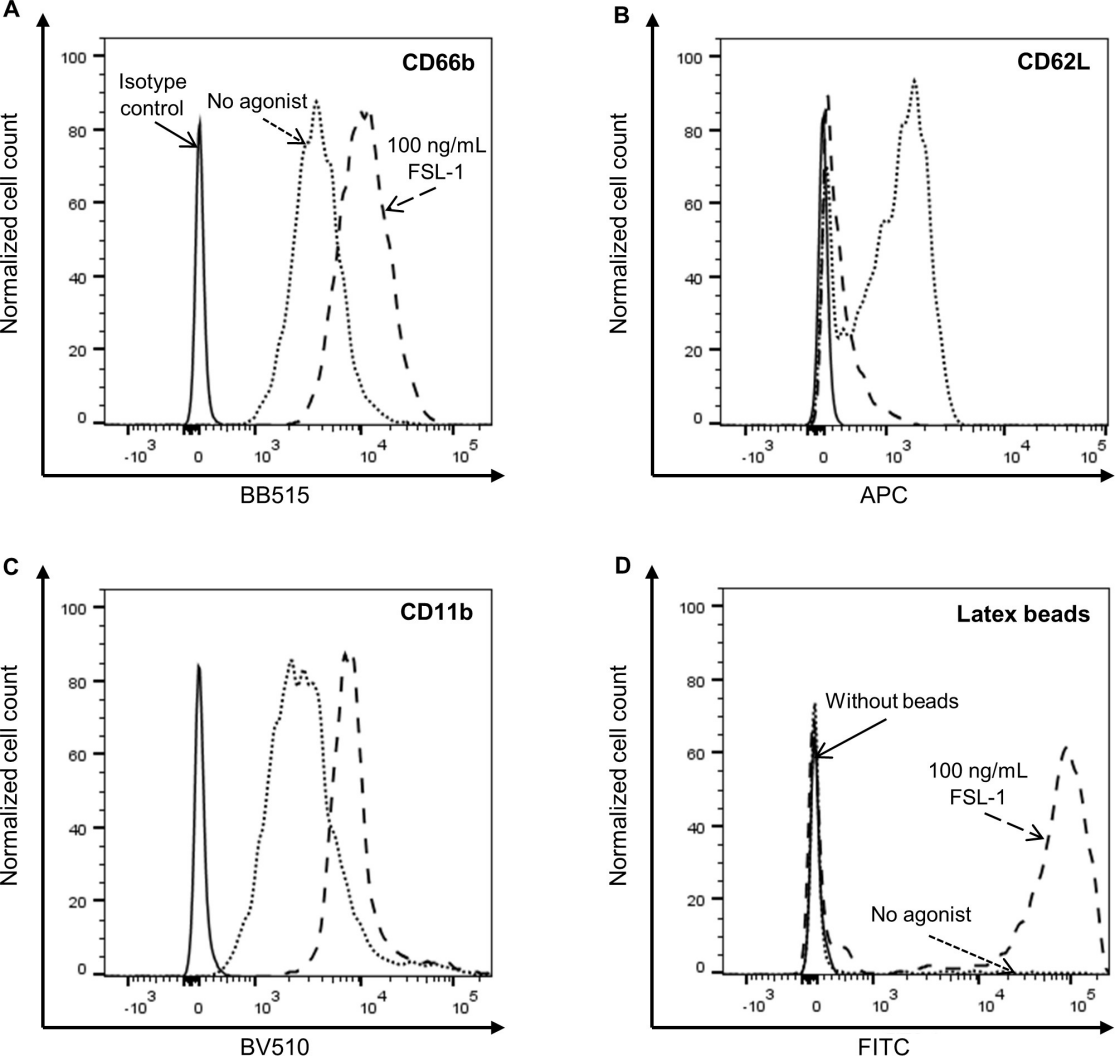
Detection of neutrophil responses to *in vitro* TLR stimulation by flow cytometry. Highly CD16-positive neutrophils were identified as outlined in S2 Fig. Representative plots of neutrophil CD66b expression (A), CD62L expression (B) and CD11b expression (C) are shown for unstimulated and FSL-1 (100 ng/mL)-stimulated neutrophils following 4 hours of culture. CD66b and CD11b expression levels increase following neutrophil activation, while CD62L levels decrease (CD62L is shed from the neutrophil surface). A representative plot of neutrophil phagocytic activity, as measured by the percentage of latex bead-FITC-positive neutrophils (indicating % of neutrophils that have internalized latex beads conjugated to FITC), is shown for unstimulated and FSL-1-stimulated (100 ng/mL) conditions (D).

**Fig 2:**
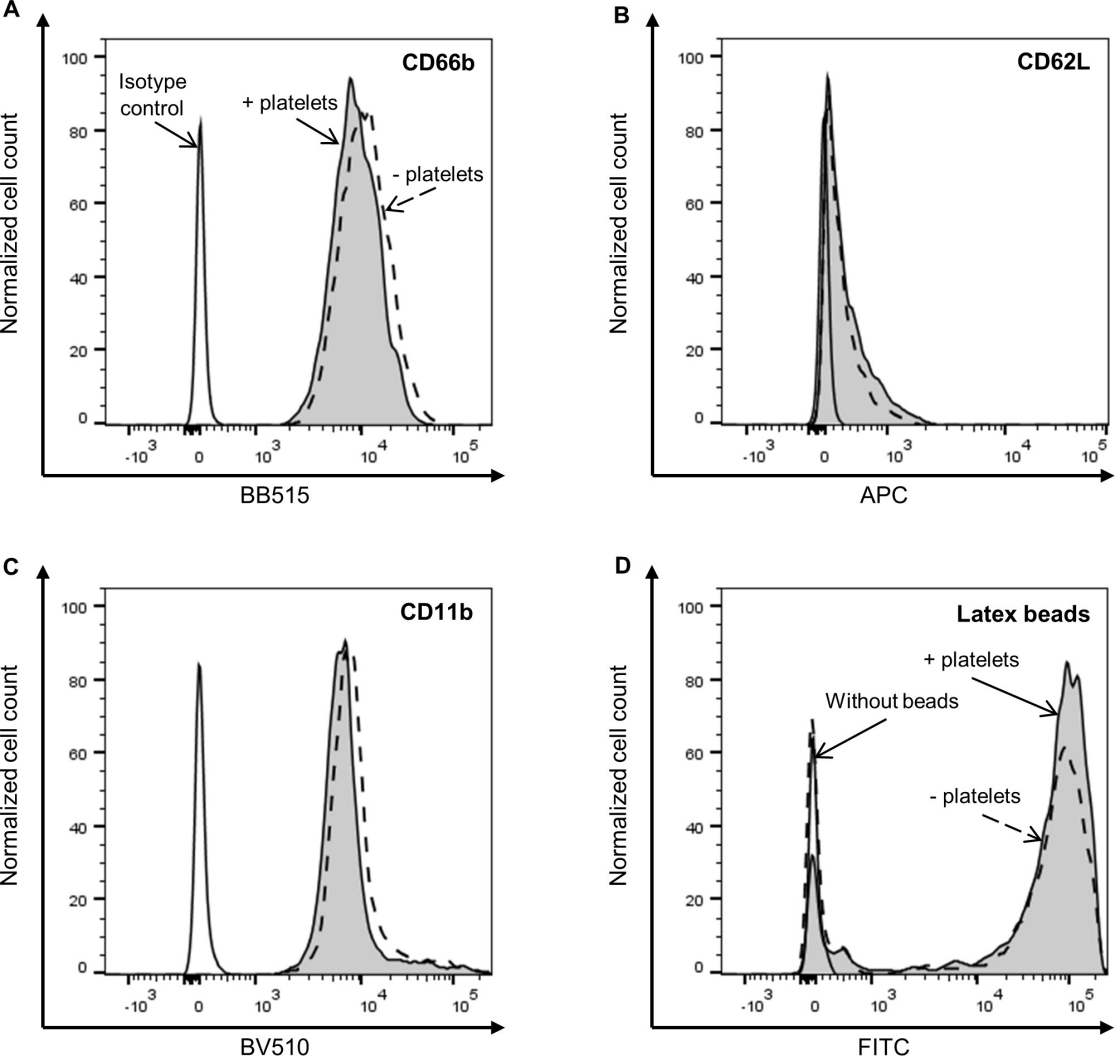
Platelets modulate neutrophil responses to *in vitro* TLR stimulation. Representative plots of neutrophil CD66b expression (A), CD62L expression (B), CD11b expression (C), and neutrophil phagocytic activity (D) are shown for neutrophils stimulated with 100 ng/mL FSL-1, with and without the addition of platelets.

For measuring neutrophil activation, neutrophils were identified firstly by doublet exclusion and secondly by high expression of CD16, as outlined in S2 Fig. Representative plots of each flow cytometry activation marker (CD66b, CD62L and CD11b) in unstimulated and 100 ng/mL FSL-1 stimulated cultures are given in Fig 1. Increased cell-surface CD66b and CD11b expression, and increased CD62L shedding (reduced CD62L expression) are all established markers of neutrophil activation [28, 29]. For reporting on the expression of CD11b, CD66b and CD62L, delta geometric mean fluorescence intensity (ΔgMFI) was calculated by subtracting the gMFI of the isotype control from the gMFI of each sample for each antibody used. For measuring neutrophil phagocytosis, neutrophils were identified by doublet exclusion. The percentage of neutrophils that were positive for FITC fluorescence (positive for internalizing latex beads conjugated to FITC) was reported. A representative plot of neutrophil phagocytosis is given in Fig 1. Representative plots of these flow cytometry markers (CD11b, CD66b, CD62L, phagocytosis) with and without the addition of platelets in 100 ng/mL FSL-1 stimulated cultures are given in Fig 2. Platelets become dimly positive for PAC1, a platelet activation marker, following incubation with these TLR agonists (without the addition of neutrophils), and expression increases when platelets and neutrophils are co-cultured together (S3 Fig).

Elastase secretion (Abcam, Cambridge, United Kingdom) and IL-8 secretion (Thermo Fisher Scientific, Waltham, MA, USA) was measured by ELISA, as per the manufacturers’ instructions.

### Statistical analysis

Continuous variables are expressed as mean ± standard deviation. In the absence of platelets, the changes in markers of neutrophil function in response to TLR stimulation were examined using one-way ANOVA with post-hoc Dunnett multiple comparison tests. To examine the effect of platelets on the neutrophil response to TLR stimulation, each baseline (- platelets) neutrophil only measurement was normalized to 1 and each platelet co-culture (+ platelets) measurement was reported as a relative change. Differences in these relative changes (- platelets vs. + platelets) were examined using a repeated measures two-way ANOVA (row factor as culture condition, column factor as ± platelets) with post-hoc Sidak multiple comparison tests (to compare row means across the column factor) using GraphPad Prism 7 (GraphPad Software Inc.).

## Results

### Platelets differentially modulate markers of neutrophil activation in response to TLR stimulation

**Fig 3:**
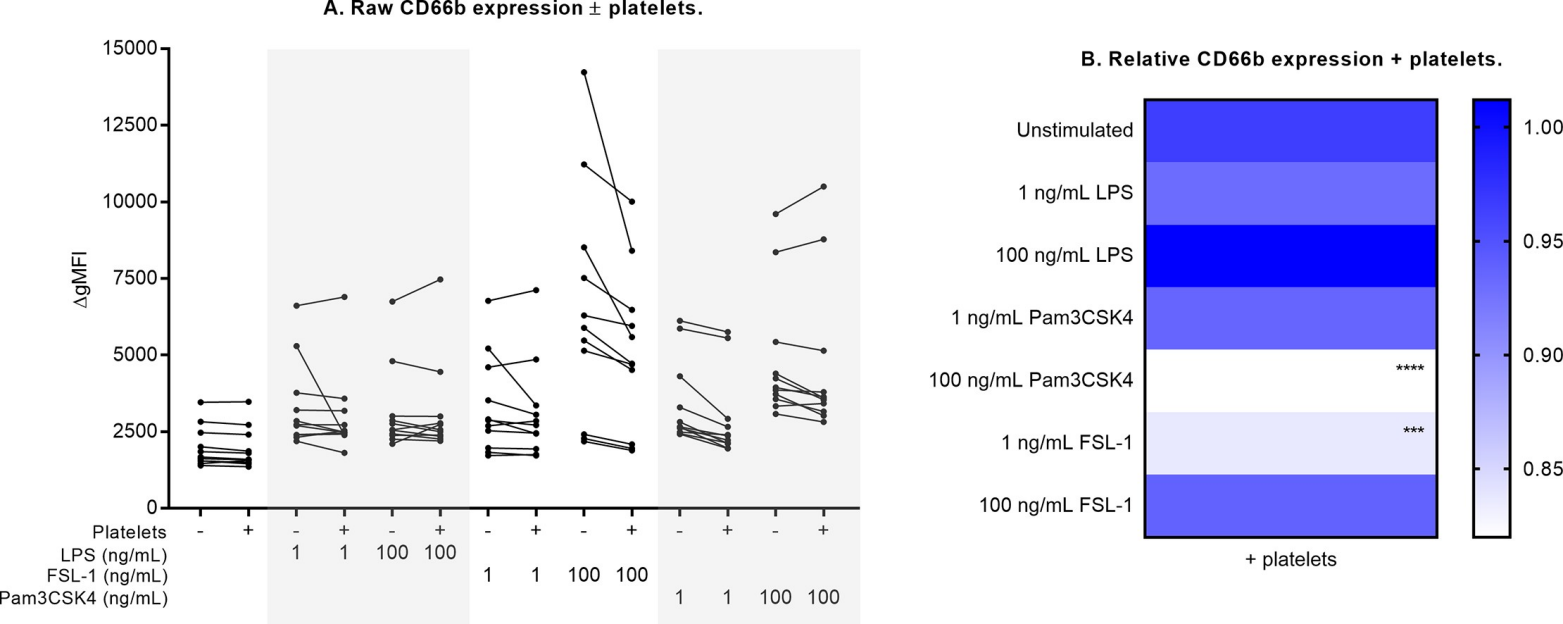
Neutrophil CD66b expression ± platelet co-culture. Raw CD66b expression results are shown in (A). CD66b was measured as ΔgMFI without platelets (- platelets) and with platelets (+ platelets) in unstimulated or TLR-stimulated cultures. For each culture condition, paired measurements (- platelets vs. + platelets) are linked with a solid black line for n = 10 healthy subjects. Relative change in the presence of platelets is shown in (B). ΔgMFI in neutrophil only cultures (- platelets) was normalized to 1 (ble) and each co-culture measurement (+ platelets) was compared to this normalized response. Differences between expression with and without platelets were examined by repeated measures two-way ANOVA with post-hoc Sidak multiple comparisons tests. ***p<0.001, ****p<0.0001.

**Fig 4:**
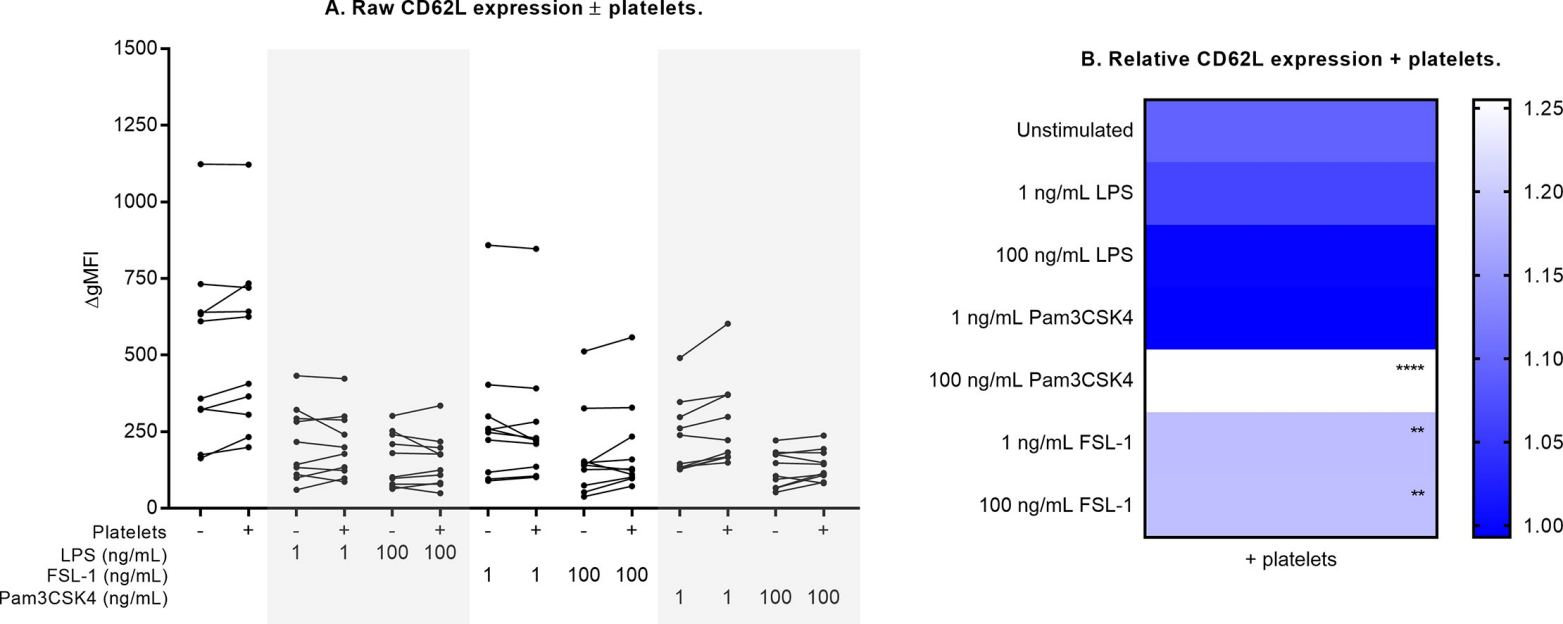
Neutrophil CD62L expression ± platelet co-culture. Raw CD62L expression results are shown in (A) for each culture condition, with each paired measurement (- platelets vs. + platelets) linked with a solid black line. CD62L expression is expressed on resting neutrophils, and is shed from the neutrophil surface in response to stimulation. Relative change in the presence of platelets is shown in (B), compared against measurements from neutrophil-only cultures, which were normalized to 1 (ble). Differences in expression (- platelets vs. + platelets) were examined by repeated measures two-way ANOVA with post-hoc Sidak multiple comparisons tests. **p<0.01, ****p<0.0001.

**Fig 5:**
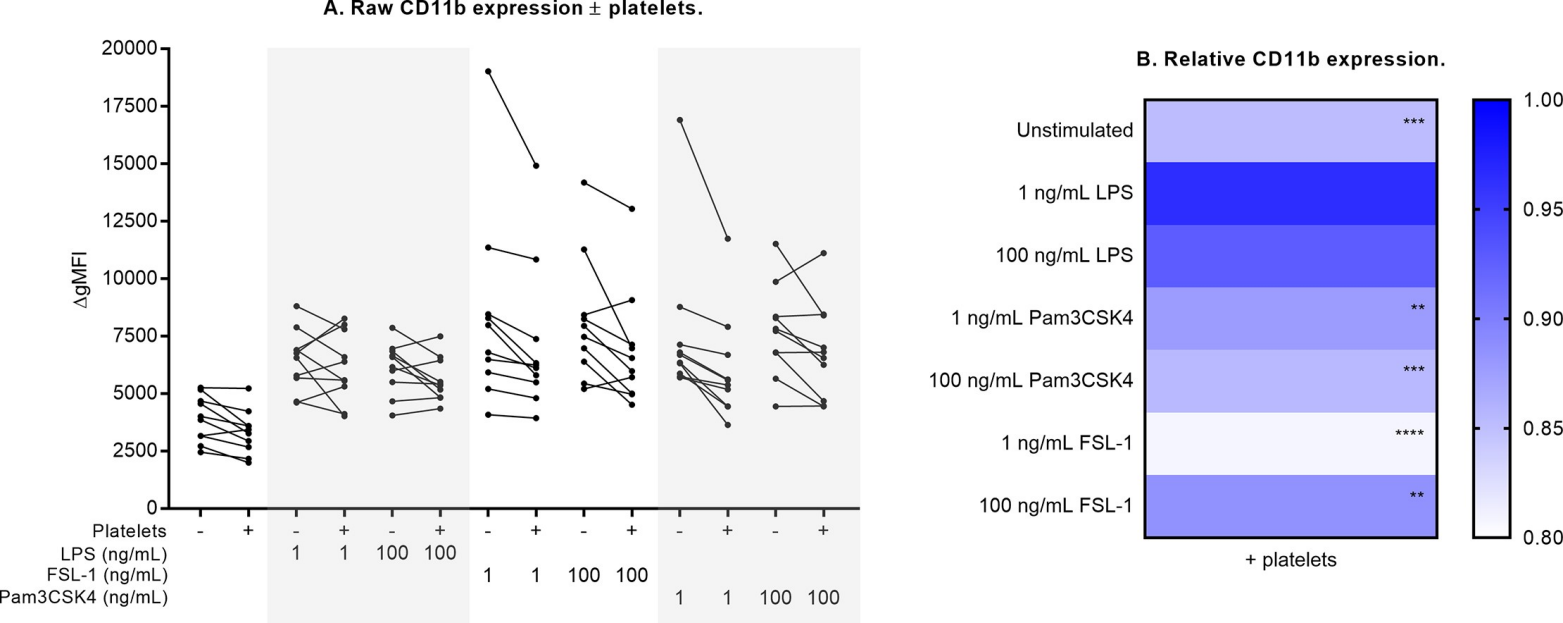
Neutrophil CD11b expression ± platelet co-culture. Raw CD11b expression results are shown in (A) for each culture condition, with each paired measurement (- platelets vs. + platelets) linked with a solid black line. Relative change in the presence of platelets is shown in (B), compared against measurements from neutrophil-only cultures normalized to 1 (ble). Differences in expression (- platelets vs. + platelets) were examined by repeated measures two-way ANOVA with post-hoc Sidak multiple comparisons tests. **p<0.01, ***p<0.001, ****p<0.0001.

In the absence of platelets, stimulation with each TLR agonist induced a significant increase in neutrophil CD66b and CD11b expression and a significant decrease in CD62L expression (indicating increased CD62L shedding). Expression of these markers under each culture condition is shown in Figs 3–5(A), and is tabulated in S1, S2 and S3 Tables. Incubation with 1 and 100 ng/mL of each TLR agonist represented sub-maximal and maximal stimulation, respectively. In response to TLR stimulation, the addition of platelets differentially affected these expression levels (Figs 3–5(B), Table 1). All three markers of neutrophil activation (CD66b and CD11b expression, CD62L shedding) measured in this study were attenuated by platelets, and were sensitive to modulation by platelets only in response to Pam3CSK4 and FSL-1, but not in response to LPS.

**Table 1:** Relative change in neutrophil markers measured by flow cytometry in neutrophil-platelet co-culture.

		+ platelets^1^			
Agonist	ng/mL	CD66b	CD62L	CD11b	Phagocytosis
Unstimulated	--	0.97 ± 0.04	1.09 ± 0.14	0.85 ± 0.13***	2.70 ± 1.10****
LPS	1	0.92 ± 0.17	1.06 ± 0.27	0.96 ± 0.19	1.08 ± 0.15
	100	1.00 ± 0.12	1.00 ± 0.21	0.93 ± 0.14	1.03 ± 0.12
Pam3CSK4	1	0.94 ± 0.12	0.99 ± 0.14	0.88 ± 0.09**	1.78 ± 0.76****
	100	0.82 ± 0.11****	1.25 ± 0.42****	0.85 ± 0.16***	1.17 ± 0.33
FSL-1	1	0.84 ± 0.09***	1.19 ± 0.13**	0.81 ± 0.12****	1.41 ± 0.31*
	100	0.94 ± 0.10	1.19 ± 0.35**	0.88 ± 0.15**	1.11 ± 0.13

^1^ For each subject, all neutrophil only measurements (- platelets) were normalized to 1, and all co-culture measurements (+ platelets) were compared to this normalized response. Relative change in ΔgMFI (CD66b, CD62L, CD11b) or % phagocytosis presented as mean ± standard deviation for n = 10 healthy subjects. Differences (- platelets vs. + platelets) were examined by a repeated measures two-way ANOVA with post-hoc Sidak multiple comparisons tests.

*p<0.05

**p<0.01

***p<0.001

****p<0.0001.

Addition of platelets attenuated CD66b expression by 18% in response to high-dose (100 ng/mL) Pam3CSK4, and by 16% in response to low-dose (1 ng/mL) FSL-1 (Fig 3B, all p<0.01). With platelet co-culture, neutrophil CD62L expression was higher in response to high-dose Pam3CSK4 (25% higher, p<0.0001) and both doses of FSL-1 (19% higher, p<0.01) compared to neutrophil-only cultures (Fig 4B). These results indicate that platelets attenuate neutrophil CD62L shedding under these conditions.CD11b expression was attenuated in the presence of platelets in response to both Pam3CSK4 and FSL-1 (12% to 19% reduction across these culture conditions, all p<0.01, Fig 5B). Platelets also modulated CD11b expression in unstimulated cultures (15% reduction, p<0.001).

### Platelets enhance neutrophil phagocytosis in unstimulated cultures and in response to some TLR agonists

**Fig 6:**
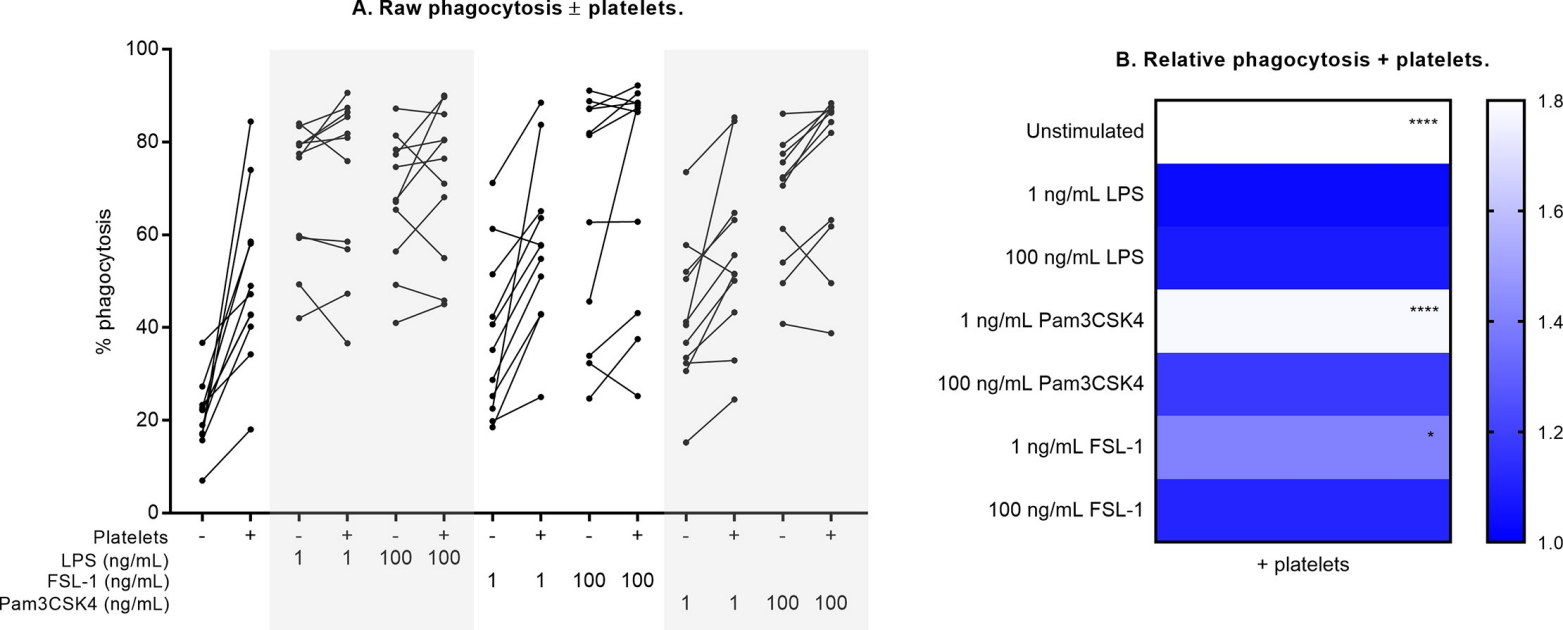
Neutrophil phagocytosis ± platelet co-culture. Phagocytosis was measured as the % of the neutrophil population that was FITC-positive (positive for internalizing latex beads conjugated to FITC) under each culture condition. Raw phagocytosis results are shown in (A). Relative change in the presence of platelets is shown in (B), compared against measurements from neutrophil-only cultures normalized to 1 (ble). Differences in expression (- platelets vs. + platelets) were examined by repeated measures two-way ANOVA with post-hoc Sidak multiple comparisons tests. *p <0.05, ****p<0.0001.

Compared to unstimulated cultures, baseline neutrophil phagocytic activity (in the absence of platelets) was significantly increased in response to all TLR agonist conditions (Fig 6A, S4 Table). In this study, phagocytosis was defined as the percentage of neutrophils that were positive for FITC fluorescence, indicating internalization of latex beads conjugated to FITC that were added to all neutrophil cultures. In unstimulated cultures, the percentage of neutrophils that were positive for phagocytosis was 2.7-fold higher with the addition of platelets, compared to neutrophils alone (p<0.0001, Fig 6B, Table 1). Platelet co-culture also resulted in a higher rate of phagocytosis seen in response to low-dose Pam3CSK4 (1.78-fold higher) and low-dose FSL-1 (1.41-fold higher, both p<0.05, Fig 6B).

### Platelets modulate elastase and IL-8 secretion by neutrophils

**Fig 7:**
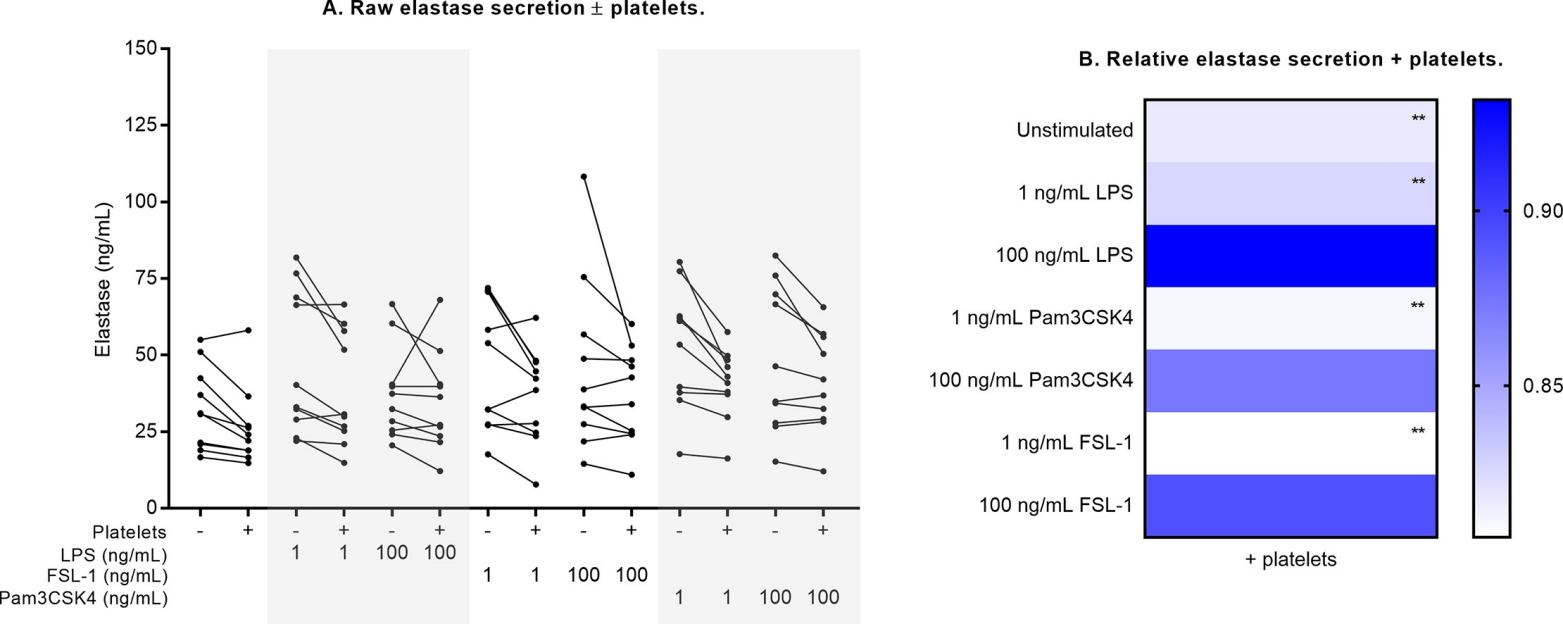
Neutrophil elastase secretion ± platelet co-culture. Raw elastase measurements are shown in (A) for each culture condition. Relative change in the presence of platelets is shown in (B), compared against measurements from neutrophil-only cultures normalized to 1 (ble). Differences in expression (- platelets vs. + platelets) were examined by repeated measures two-way ANOVA with post-hoc Sidak multiple comparisons tests. **p<0.01.

**Fig 8:**
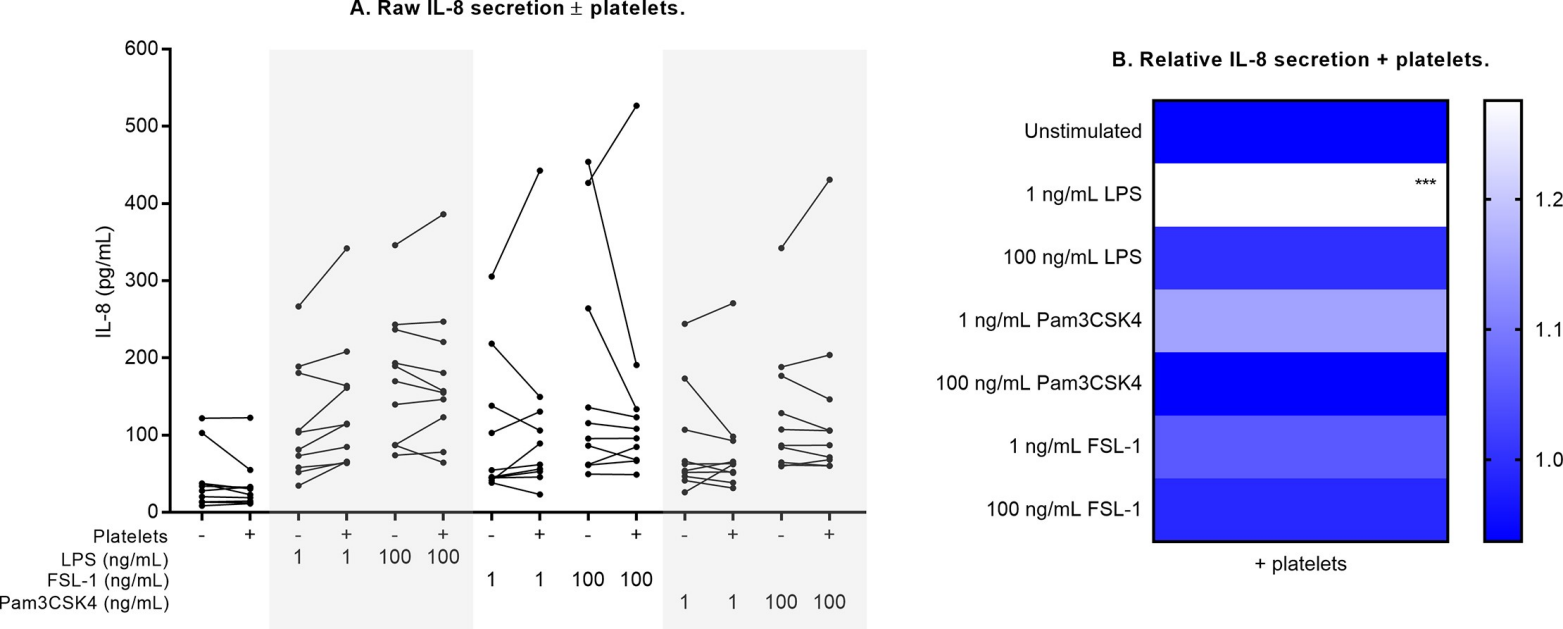
Neutrophil IL-8 secretion ± platelet co-culture. Raw IL-8 measurements are shown in (A) for each culture condition. Relative change in the presence of platelets is shown in (B), compared against measurements from neutrophil-only cultures normalized to 1 (ble). Differences in expression (- platelets vs. + platelets) were examined by repeated measures two-way ANOVA with post-hoc Sidak multiple comparisons tests. ***p<0.001.

Both elastase and IL-8 secretion were assessed from the supernatant of neutrophil-only and neutrophil-platelet co-cultures. Baseline secretion (-platelets) of both markers increased in response to TLR stimulation (Fig 7A and Fig 8A, S5 and S6 Tables). For elastase, the increase in secretion was only statistically significant in response to low-dose (1 ng/mL) TLR stimulation.

With the addition of platelets, elastase secretion was significantly lowered by 18% in unstimulated cultures (p<0.01, Fig 7B, Table 2), and the increase in elastase secretion following low-dose TLR stimulation was also attenuated by platelets (17% to 19% reduction; all p<0.01). The presence of platelets significantly altered IL-8 secretion only in response to low-dose LPS: the increase in IL-8 secretion with this dose was 1.28-fold greater in platelet co-culture (p<0.001, Fig 8B, Table 2).

**Table 2:** Relative change in neutrophil secretory products in neutrophil-platelet co-culture.

		+ platelets^1^	
Agonist	ng/mL	Elastase	IL-8
Unstimulated	--	0.82 ± 0.13**	0.94 ± 0.24
LPS	1	0.83 ± 0.14**	1.28 ± 0.28***
	100	0.93 ± 0.31	1.00 ± 0.18
Pam3CSK4	1	0.81 ± 0.23**	1.15 ± 0.45
	100	0.87 ± 0.19	0.94 ± 0.30
FSL-1	1	0.81 ± 0.13**	1.05 ± 0.51
	100	0.89 ± 0.13	0.99 ± 0.14

^1^ For each subject, all neutrophil only measurements (- platelets) were normalized to 1, and all co-culture measurements (+ platelets) were compared to this normalized response. Relative change in secretory products presented as mean ± standard deviation for n = 10 healthy subjects. Differences (- platelets vs. + platelets) were examined by a repeated measures two-way ANOVA with post-hoc Sidak multiple comparisons tests.

*p<0.05

**p<0.01

***p<0.001.

## Discussion

We demonstrate that platelets differentially modulate several neutrophil functions in a TLR agonist-specific and dose-specific manner. Furthermore, platelets can attenuate some, and simultaneously augment other, neutrophil functions. A summary of the results described in this study is given in Table 3. The influence of platelets on the expression of three well-characterized flow cytometric markers of neutrophil activation were assessed in this study. Platelets attenuated the expression of both CD66b and CD11b on neutrophils stimulated with Pam3CSK4 and FSL-1 and, additionally, CD11b expression was also attenuated in unstimulated cultures. In response to these TLR agonists, neutrophil CD62L expression was higher in the presence of platelets, indicating reduced CD62L shedding under these culture conditions. Conversely, neutrophil phagocytosis was significantly higher in co-culture both without stimulation and with TLR stimulation (Pam3CSK4 and FSL-1). Platelets did not alter the expression of any of these markers in response to LPS. We also assessed the effect of platelets on two neutrophil secretory products. Elastase secretion was attenuated in the presence of platelets in unstimulated cultures as well as in response to low-dose stimulation with all three TLR agonists. We also show that the increase in IL-8 secretion in response to low-dose LPS was further increased in the presence of platelets. In conclusion, platelets modify a number of neutrophil functions in both the absence of stimulation and in response to TLR stimulation. Additionally, it is interesting to note that platelets have a dampening effect on a majority of the markers examined here (CD66b and CD11b expression, CD62L shedding and elastase secretion).

**Table 3:** Summary of the effect of platelets on the markers of neutrophil function assessed in this study.

		+ platelets					
Agonist	ng/mL	CD66b	CD62L	CD11b	Phagocytosis	Elastase	IL-8
Unstimulated	--			↓	↑	↓	
LPS	1					↓	↑
	100						
Pam3CSK4	1			↓	↑	↓	
	100	↓	↑	↓			
FSL-1	1	↓	↑	↓	↑	↓	
	100		↑	↓			

Red shaded/↓, relative change was statistically significantly decreased with platelets; Blue shaded/↑, relative change in marker was statistically significantly increased with the addition of platelets.

This study was conducted to further investigate the findings of our previous study [16], where we demonstrated that platelets regulate a number of leukocyte responses to TLR stimulation. Here, we have expanded on the number of neutrophil functions examined, and demonstrate that platelets are able to exert both stimulatory and dampening effects on these functions. These results contribute to a growing body of evidence indicating that platelets play a dual role in inflammation. While platelets have traditionally been considered pro-inflammatory [21, 30], a number of studies [15, 16, 27, 31–34] indicate that platelets can limit inflammation by dampening leukocyte-mediated pro-inflammatory processes. Platelets are postulated to provide these cues to limit host damage that can occur during excessive inflammation, such as during sepsis [11]. Thus, it is likely that the context plays an important role in determining the platelet response during inflammation.

The activation of neutrophils is widely associated with changes in expression of CD66b, CD11b and CD62L [28, 29], and these markers function across a number of neutrophil responses including degranulation, transmigration and phagocytosis [35–38]. We show, here, that platelets can attenuate CD66b expression and this is consistent with our previous work [16], where we show that platelets reduced CD66b expression (relative reductions of 14–19%) in response to stimulation with Pam3CSK4 and FSL-1. We also show that platelets similarly dampen neutrophil CD11b expression and CD62L shedding. These results are in agreement with the findings of Corken et al. [39], who demonstrated that the loss of a single platelet receptor, GPIb-IX, was sufficient to increase the level of neutrophil Mac-1 (CD11b/CD18) expression that occurs 24 hours post onset of sepsis in a mouse model of polymicrobial sepsis.

The expression of all three markers remained unchanged by platelets in response to LPS; platelets were only able to significantly alter neutrophil activation in response to Pam3CSK4 and FSL-1. It interesting to consider what drives a difference in the magnitude of the platelet effect in response to these TLR agonists. As both Pam3CSK4 and FSL-1 signal through TLR2 heterodimers, while LPS signals through TLR4, this may be due to differential platelet-TLR signal transduction. Platelet-TLR signal transduction has been most well-studied in response to Pam3CSK4, and platelet-TLR2/1 signalling has shown to induce Src- and Syk- family kinases [40, 41], which mimics the signalling pathway of common haemostatic platelet receptors [42]. It may be that platelets can exert greater effects on neutrophils following stimulation with TLR2 agonists due to the ability to recruit or mimic haemostatic receptor pathways. However, comparing the differences in signalling induced by each TLR agonist has yet to be determined, and is an outstanding question in this field.

We show that platelets can enhance neutrophil phagocytosis both with and without TLR stimulation (low doses of Pam3CSK4 and FSL-1). These observations are consistent with prior reports: phagocytosis of periodontopathogens by neutrophils can be enhanced by platelets [43], and neutrophils within platelet-neutrophil complexes have increased phagocytic activity when compared to neutrophils that did not bind to platelets [26]. In our study, platelets did not further increase phagocytic activity under any other stimulation condition (high-dose TLR stimulation and low-dose LPS stimulation). In the absence of platelets, phagocytosis under these culture conditions was uniformly increased (65–70% of neutrophils were FITC+). One explanation for the absence of a platelet response here may be that the ‘maximum’ phagocytic activity seen under these conditions limits the ability of platelets to further enhance this activity.

Within this study, we also assessed the effect of platelets on elastase and IL-8 secretion by neutrophils. The platelet effect seen in response to low-dose TLR stimulation that we report in this study mirrors the results from our previous work [16], and is also in agreement with others [26]. Elastase is a potent serine protease which can degrade a multitude of plasma and extracellular matrix proteins. As such, extracellular elastase is a powerful bacterial killing mechanism [44] but has also been implicated in acute and chronic inflammatory host damage [45, 46]. Platelets may dampen elastase secretion as a mechanism to prevent inflammatory host damage. Conversely, platelets modulated IL-8 secretion only in response to low-dose LPS. IL-8 is an important pro-inflammatory chemoattractant protein [47] and it may be that, under particular stimulation conditions, platelets can increase neutrophil IL-8 secretion and enhance further neutrophil chemoattraction to the site of inflammation. However, it is unclear why an effect of platelets was only observed under this condition. Examining a larger panel of cytokines and chemokines secreted is required to contextualize the significance of this platelet effect on IL-8 secretion.

The combination of the platelet effects described in this study suggest that platelets can both augment and attenuate neutrophil functions, and modulation of these functions can occur in both the absence and presence of TLR stimulation. For example, we show that platelets can simultaneously reduce elastase secretion and increase phagocytic activity in unstimulated cultures. Similarly, these opposing effects are seen following stimulation with Pam3CSK4 and FSL-1. The opposing effect of platelets noted here may be a mechanism for enhancing particular anti-microbial functions (for example, phagocytosis) while dampening neutrophil functions that are more likely to lead to host damage, if unchecked (for example, elastase secretion). Overall, we suggest that platelets finely regulate neutrophil functions, rather than providing broad anti-inflammatory cues to these cells, and we postulate that the platelet effect can modulate some aspects of the inflammatory environment to reduce host damage.

We also note that the platelet effect on these markers of neutrophil function was modulatory, rather than completely inhibitory. Neutrophils are highly reactive and short-lived cells that are crucial to anti-microbial host defence, but have also been implicated in causing inflammatory host damage [48, 49]. Therefore, the inflammatory response must be rapid and robust, but also must be tightly controlled to prevent rampant inflammation. We suggest that platelets are regulators of neutrophil function and, within this role, platelets can act as a brake to neutrophil-mediated inflammation. In this regard, the platelet effect can be considered a mechanism for inflammation control.

We speculate, here, on how these results can be interpreted in the context of clinical pathologies that are characterized by platelet activation and inflammation. The newly-emerging role of platelets as immune regulators in sepsis [11], cardiac ischaemia/reperfusion (I/R) injury [17, 20] and acute lung injury (ALI) [50] is often juxtaposed against their well-established role in promoting leukocyte-mediated inflammation in these inflammatory diseases. An emerging working hypothesis is that platelets can elicit dual, potentially sequential, roles in inflammation [11, 20]. Platelets can enhance leukocyte infiltration and inflammation to provide anti-microbial protection during infection or to restore haemostasis during sterile inflammation. Furthermore, platelets can then switch their phenotype to temper this inflammatory response to protect from host damage.

In the context of sepsis and septic shock, there has been particular focus on how platelets modulate their cytokine environment. Thrombocytopenia in septic patients [51] and platelet depletion in mouse models of sepsis [18, 52] is associated with mortality, and the loss of platelets can drive an elevation in plasma pro-inflammatory cytokines (TNFα and IL-6) in septic mice [18, 53, 54]. We also add that, in this study, platelets can dampen neutrophilic inflammation in a stimulus-specific manner. Conversely, unregulated NET formation, propagated by platelet-neutrophil interactions [2], can cause significant host damage during sepsis by initiating intravascular thrombosis [24, 55]. To add complexity, NETs have also been shown to degrade cytokines and chemokines and, consequently, reduce inflammation [56].

In a similar vein, platelets can exacerbate [23, 57] and protect against [58, 59] ALI. Platelets have been shown to mediate these opposing effects via their interaction with neutrophils. For example, platelet-neutrophil cross-talk is essential to early inflammatory responses in the lung [23, 57], but these aggregates can also specifically produce and process the pro-resolving mediator, Maresin 1, to reduce lung inflammation [59]. The ability of platelets to propagate and resolve inflammation is particularly important in acute myocardial infarction (AMI) and I/R injury, where the resolution of inflammation in a timely manner is required for optimal myocardial healing and long-term repair [60]. In both conditions, platelets are known to have both pro-inflammatory [17, 61] and pro-resolving [20, 62] effects. In a clinical setting, anti-platelet agents are administered as a cornerstone of intervention in AMI. Although necessary to inhibit the pro-thrombotic effects of platelets during AMI, this treatment strategy may inhibit other, more subtle and perhaps beneficial, platelet immune effects. For example, the ability of platelets to control human neutrophil production of reactive oxygen species is reduced by administration of aspirin or clopidogrel [15]. In a similar vein, the platelet effect on neutrophil function that we report in this study may also be abolished by potent anti-platelet therapy.

To summarize, the immune functions of platelets are various and diverse. It is likely that the induction of a particular platelet response is a combination of the type of ligand present, the magnitude of the immune response and the type of platelet-leukocyte interaction occurring. In particular, platelets are known to elicit different responses to various agonists [63, 64], which includes the differential release of immunomodulators from their stored granules [65, 66]. This suggests an ability to elicit distinct pro-inflammatory or anti-inflammatory responses depending on the type of inflammatory stimulus received by the platelet population.

Our study had a number of limitations. The methodology was designed to minimize neutrophil activation prior to TLR stimulation, but we cannot be certain that the activation state was not altered by the isolation process. Neutrophils and platelets do not act in isolation and it may be that, within whole blood, the modulation of neutrophil function by platelets differs Additionally, although Pam3CSK4 shows 98.1% purity, we cannot guarantee that this reagent did not contain any contaminants with TLR agonist activity. Finally, we did not assess whether the effect of platelets was directly or indirectly mediated, rather we aimed to examine the holistic platelet effects in this study.

## Conclusion

In conclusion, the combination of the platelet effects described in this study suggest that platelets can both augment and attenuate neutrophil functions, and modulation of these functions can occur in both the absence and presence of TLR stimulation. We suggest that platelets regulate neutrophil function in a complex manner, rather than providing broad anti-inflammatory cues to these cells. This platelet effect may modulate some aspects of the inflammatory environment to reduce host damage.

## Supporting information

S1 Fig Platelets remain quiescent both pre- and post-culture. Platelets were isolated and probed for activation post-isolation and prior to culture (pre-culture; dashed line). Platelets were then cultured alone in the absence of TLR stimulation for 4 hours (post-culture; solid + filled line), and also probed for activation. To detect activation, platelets were incubated with PAC1-FITC (clone PAC-1, Becton Dickinson) for 30 minutes at 4°C, fixed and analysed. PAC1 expression under both conditions was minimal, when compared to unstained platelets (dot-dashed line). PAC1 is a common platelet activation marker that recognizes the activation-dependent glycoprotein IIb/IIIa complex on the platelet surface.
(TIF)

S2 Fig Gating strategy to identify neutrophils after 4-hour culture. Initially, all samples were visualised by SSC-A vs. time to check the flow of cells during acquisition. Doublet exclusion was performed firstly by examining the SSC-A vs. SSC-H profile (A) and secondly by examining the FSC-A vs. FSC-H profile (B). The resulting SSC-A vs. FSC-A profile of the population is shown in (C). Neutrophils were identified as cells that were highly expressing CD16 (D) against the isotype control (E). These representative plots are from a sample of unstimulated neutrophils, and we did not observe a visual difference between unstimulated and stimulated neutrophils using these plots.
(TIF)

S3 Fig Platelets become dimly positive for PAC1 following incubation with TLR agonists. Platelets were isolated and cultured alone (dotted line) or with neutrophils (dashed line) for 4 hours in the presence of 100 ng/mL of either LPS (A), Pam3CSK4 (B) or FSL-1 (C). Platelets were stained for PAC1-FITC as described in S2 Fig. PAC1 expression under these stimulation conditions was compared to unstimulated platelets (solid + filled line). The shift in PAC1 expression with TLR stimulation was more pronounced for LPS and FSL-1, compared to Pam3CSK4. The same trend was observed when platelets were stimulated with 1 ng/mL of all TLR agonists (data not shown).
(TIF)

S1 Table Raw neutrophil CD66b expression ± platelet co-culture. (TIF)

S2 Table Raw neutrophil CD62L expression ± platelet co-culture. (TIF)

S3 Table Raw neutrophil CD11b expression ± platelet co-culture. (TIF)

S4 Table Raw neutrophil phagocytosis ± platelet co-culture. (TIF)

S5 Table Raw neutrophil elastase secretion ± platelet co-culture. (TIF)

S6 Table Raw neutrophil IL-8 secretion ± platelet co-culture. (TIF)

## Abbreviations

FSL-1: Fibroblast-stimulating lipopeptide -1
LPS: Lipopolysaccharide
NETs: Neutrophil extracellular traps
PPP: Platelet-poor plasma
PRP: Platelet-rich plasma
ROS: Reactive oxygen species
TLR: Toll-like receptors
WPs: Washed platelets

## References

[1] Yeaman MR. Platelets: at the nexus of antimicrobial defence. *Nature Reviews Microbiology*. 2014; 12: 426–37. 10.1038/nrmicro3269 24830471

[2] Clark SR, Ma AC, Tavener SA, McDonald B, Goodarzi Z, Kelly MM, et al Platelet TLR4 activates neutrophil extracellular traps to ensnare bacteria in septic blood. *Nat Med*. 2007; 13: 463–9. 10.1038/nm1565 17384648

[3] Verschoor A, Neuenhahn M, Navarini AA, Graef P, Plaumann A, Seidlmeier A, et al A platelet-mediated system for shuttling blood-borne bacteria to CD8[alpha]+ dendritic cells depends on glycoprotein GPIb and complement C3. *Nat Immunol*. 2011; 12: 1194–201. 10.1038/ni.2140 22037602

[4] Cognasse F, Hamzeh H, Chavarin P, Acquart S, Genin C, Garraud O. Evidence of Toll-like receptor molecules on human platelets. *Immunol Cell Biol*. 2005; 83: 196–8. 10.1111/j.1440-1711.2005.01314.x 15748217

[5] Shiraki R, Inoue N, Kawasaki S, Takei A, Kadotani M, Ohnishi Y, et al Expression of Toll-like receptors on human platelets. *Thromb Res*. 2004; 113: 379–85. 10.1016/j.thromres.2004.03.023 15226092

[6] Hally KE, La Flamme AC, Larsen PD, Harding SA. Platelet Toll-like receptor (TLR) expression and TLR-mediated platelet activation in acute myocardial infarction. *Thromb Res*. 2017; 158: 8–15. 10.1016/j.thromres.2017.07.031 28783513

[7] Blair P, Rex S, Vitseva O, Beaulieu L, Tanriverdi K, Chakrabarti S, et al Stimulation of Toll-like receptor 2 in human platelets induces a thromboinflammatory response through activation of phosphoinositide 3-kinase. *Circ Res*. 2009; 104: 346–54. 10.1161/CIRCRESAHA.108.185785 19106411 PMC2732983

[8] Rivadeneyra L, Carestia A, Etulain J, Pozner RG, Fondevila C, Negrotto S, et al Regulation of platelet responses triggered by Toll-like receptor 2 and 4 ligands is another non-genomic role of nuclear factor-kappaB. *Thromb Res*. 2014; 133: 235–43. 10.1016/j.thromres.2013.11.028 24331207

[9] Zhang G, Han J, Welch EJ, Ye RD, Voyno-Yasenetskaya TA, Malik AB, et al Lipopolysaccharide stimulates platelet secretion and potentiates platelet aggregation via TLR4/MyD88 and the cGMP-dependent protein kinase pathway. *J Immunol*. 2009; 182: 7997–8004. 10.4049/jimmunol.0802884 19494325 PMC2787095

[10] Hally KE, La Flamme AC, Harding SA, Larsen PD. The effects of aspirin and ticagrelor on Toll-like receptor (TLR)-mediated platelet activation: results of a randomized, cross-over trial. *Platelets*. 2018: 1–9. 10.1080/09537104.2018.1479520 29869943

[11] Stocker TJ, Ishikawa-Ankerhold H, Massberg S, Schulz C. Small but mighty: Platelets as central effectors of host defense. *Thromb Haemost*. 2017; 117: 651–61. 10.1160/TH16-12-0921 28203681

[12] Ståhl A-l, Svensson M, Mörgelin M, Svanborg C, Tarr PI, Mooney JC, et al Lipopolysaccharide from enterohemorrhagic Escherichia coli binds to platelets through TLR4 and CD62 and is detected on circulating platelets in patients with hemolytic uremic syndrome. *Blood*. 2006; 108: 167–76. 10.1182/blood-2005-08-3219 16514062 PMC1895830

[13] Linke B, Schreiber Y, Picard-Willems B, Slattery P, Nusing RM, Harder S, et al Activated Platelets Induce an Anti-Inflammatory Response of Monocytes/Macrophages through Cross-Regulation of PGE2 and Cytokines. *Mediators Inflamm*. 2017; 2017: 1463216 10.1155/2017/1463216 28592915 PMC5448075

[14] Jancinova V, Drabikova K, Petrikova M, Nosal R. Blood platelets decrease concentration of reactive oxygen species produced by polymorphonuclear leukocytes. *Bratisl Lek Listy*. 2004; 105: 250–5. 15543845

[15] Reinisch CM, Dunzendorfer S, Pechlaner C, Ricevuti G, Wiedermann CJ. The inhibition of oxygen radical release from human neutrophils by resting platelets is reversed by administration of acetylsalicylic acid or clopidogrel. *Free Radic Res*. 2001; 34: 461–6. 10.1080/10715760100300401 11378529

[16] Hally KE, La Flamme AC, Harding SA, Larsen PD. Platelets regulate leucocyte responses to Toll-like receptor stimulation. Clinical & Translational *Immunology*. 2018; 7: e1036. 30065836 10.1002/cti2.1036 PMC6063753

[17] Ziegler M, Wang X, Peter K. Platelets in cardiac ischaemia/reperfusion injury: a promising therapeutic target. *Cardiovasc Res*. 2019; 115: 1178–88. 10.1093/cvr/cvz070 30906948 PMC6529900

[18] Xiang B, Zhang G, Guo L, Li XA, Morris AJ, Daugherty A, et al Platelets protect from septic shock by inhibiting macrophage-dependent inflammation via the cyclooxygenase 1 signalling pathway. *Nature Communications*. 2013; 4: 2657 10.1038/ncomms3657 24150174 PMC4217311

[19] Bain W, Olonisakin T, Yu M, Qu Y, Hulver M, Xiong Z, et al Platelets inhibit apoptotic lung epithelial cell death and protect mice against infection-induced lung injury. *Blood Advances*. 2019; 3: 432–45. 10.1182/bloodadvances.2018026286 30733303 PMC6373758

[20] Walsh TG, Poole AW. Do platelets promote cardiac recovery after myocardial infarction: roles beyond occlusive ischemic damage. *Am J Physiol Heart Circ Physiol*. 2018; 314: H1043–h8. 10.1152/ajpheart.00134.2018 29547023 PMC6008147

[21] Gros A, Ollivier V, Ho-Tin-Noe B. Platelets in inflammation: regulation of leukocyte activities and vascular repair. *Front Immunol*. 2014; 5: 678 10.3389/fimmu.2014.00678 25610439 PMC4285099

[22] Assinger A, Laky M, Badrnya S, Esfandeyari A, Volf I. Periodontopathogens induce expression of CD40L on human platelets via TLR2 and TLR4. *Thromb Res*. 2012; 130: e73–e8. 10.1016/j.thromres.2012.04.017 22608210

[23] Zarbock A, Singbartl K, Ley K. Complete reversal of acid-induced acute lung injury by blocking of platelet-neutrophil aggregation. *J Clin Invest*. 2006; 116: 3211–9. 10.1172/JCI29499 17143330 PMC1679711

[24] McDonald B, Davis RP, Kim SJ, Tse M, Esmon CT, Kolaczkowska E, et al Platelets and neutrophil extracellular traps collaborate to promote intravascular coagulation during sepsis in mice. *Blood*. 2017; 129: 1357–67. 10.1182/blood-2016-09-741298 28073784 PMC5345735

[25] Herbertsson H, Bengtsson T. Role of platelets and the arachidonic acid pathway in the regulation of neutrophil oxidase activity. *Scand J Clin Lab Invest*. 2001; 61: 641–9. 10.1080/003655101753268008 11768324

[26] Losche W, Dressel M, Krause S, Redlich H, Spangenberg P, Heptinstall S. Contact-induced modulation of neutrophil elastase secretion and phagocytic activity by platelets. *Blood Coagul Fibrinolysis*. 1996; 7: 210–3. 10.1097/00001721-199603000-00025 8735821

[27] Del Principe D, Menichelli A, Di Giulio S, De Matteis W, Giordani M, Pentassuglio AM, et al Stimulated platelets release factor(s) affecting the in vitro response of human polymorphonuclear cells. *J Leukoc Biol*. 1990; 48: 7–14. 10.1002/jlb.48.1.7 2113564

[28] Fortunati E, Kazemier KM, Grutters JC, Koenderman L, Van den Bosch vJMM. Human neutrophils switch to an activated phenotype after homing to the lung irrespective of inflammatory disease. *Clin Exp Immunol*. 2009; 155: 559–66. 10.1111/j.1365-2249.2008.03791.x 19077082 PMC2669533

[29] van Oostrom AJ, van Wijk JP, Sijmonsma TP, Rabelink TJ, Castro Cabezas M. Increased expression of activation markers on monocytes and neutrophils in type 2 diabetes. *The Netherlands Journal of Medicine*. 2004; 62: 320–5. 15635816

[30] Stokes KY, Granger DN. Platelets: a critical link between inflammation and microvascular dysfunction. *The Journal of Physiology*. 2012; 590: 1023–34. 10.1113/jphysiol.2011.225417 22183721 PMC3381810

[31] Gudbrandsdottir S, Hasselbalch HC, Nielsen CH. Activated platelets enhance IL-10 secretion and reduce TNF-alpha secretion by monocytes. *J Immunol*. 2013; 191: 4059–67. 10.4049/jimmunol.1201103 24048901

[32] Nami N, Feci L, Napoliello L, Giordano A, Lorenzini S, Galeazzi M, et al Crosstalk between platelets and PBMC: New evidence in wound healing. *Platelets*. 2016; 27: 143–8. 10.3109/09537104.2015.1048216 26030799

[33] Sadallah S, Eken C, Martin PJ, Schifferli JA. Microparticles (ectosomes) shed by stored human platelets downregulate macrophages and modify the development of dendritic cells. *J Immunol*. 2011; 186: 6543–52. 10.4049/jimmunol.1002788 21525379

[34] Jancinova V, Drabikova K, Nosal R, Petrikova M, Ciz M, Lojek A, et al Inhibition of FMLP-stimulated neutrophil chemiluminescence by blood platelets increased in the presence of the serotonin-liberating drug chloroquine. *Thromb Res*. 2003; 109: 293–8. 10.1016/s0049-3848(03)00239-1 12818253

[35] Deree J, Lall R, Melbostad H, Grant M, Hoyt DB, Coimbra R. Neutrophil degranulation and the effects of phosphodiesterase inhibition. *J Surg Res*. 2006; 133: 22–8. 10.1016/j.jss.2006.02.031 16690368

[36] Lacy P. Mechanisms of Degranulation in Neutrophils. Allergy, Asthma, and Clinical Immunology: Official Journal of the Canadian Society of Allergy and Clinical *Immunology*. 2006; 2: 98–108. 10.1186/1710-1492-2-3-98 PMC2876182 20525154

[37] Le Cabec V, Carreno S, Moisand A, Bordier C, Maridonneau-Parini I. Complement receptor 3 (CD11b/CD18) mediates type I and type II phagocytosis during nonopsonic and opsonic phagocytosis, respectively. *J Immunol*. 2002; 169: 2003–9. 10.4049/jimmunol.169.4.2003 12165526

[38] Diacovo TG, Roth SJ, Buccola JM, Bainton DF, Springer TA. Neutrophil rolling, arrest, and transmigration across activated, surface-adherent platelets via sequential action of P-selectin and the beta 2-integrin CD11b/CD18. *Blood*. 1996; 88: 146–57. 8704169

[39] Corken A, Russell S, Dent J, Post SR, Ware J. Platelet glycoprotein Ib-IX as a regulator of systemic inflammation. *Arterioscler Thromb Vasc Biol*. 2014; 34: 996–1001. 10.1161/ATVBAHA.113.303113 24504734 PMC3991762

[40] Klarstrom Engstrom K, Brommesson C, Kalvegren H, Bengtsson T. Toll like receptor 2/1 mediated platelet adhesion and activation on bacterial mimetic surfaces is dependent on src/Syk-signaling and purinergic receptor P2X1 and P2Y12 activation. *Biointerphases*. 2014; 9: 041003 10.1116/1.4901135 25553878

[41] Falker K, Klarstrom-Engstrom K, Bengtsson T, Lindahl TL, Grenegard M. The toll-like receptor 2/1 (TLR2/1) complex initiates human platelet activation via the src/Syk/LAT/PLCgamma2 signalling cascade. *Cell Signal*. 2014; 26: 279–86. 10.1016/j.cellsig.2013.11.011 24240055

[42] Senis YA, Mazharian A, Mori J. Src family kinases: at the forefront of platelet activation. *Blood*. 2014; 124: 2013–24. 10.1182/blood-2014-01-453134 25115887 PMC4186533

[43] Assinger A, Laky M, Schabbauer G, Hirschl AM, Buchberger E, Binder BR, et al Efficient phagocytosis of periodontopathogens by neutrophils requires plasma factors, platelets and TLR2. *J Thromb Haemost*. 2011; 9: 799–809. 10.1111/j.1538-7836.2011.04193.x 21251195

[44] Belaaouaj A, Kim KS, Shapiro SD. Degradation of outer membrane protein A in Escherichia coli killing by neutrophil elastase. *Science*. 2000; 289: 1185–8. 10.1126/science.289.5482.1185 10947984

[45] Kawabata K, Hagio T, Matsuoka S. The role of neutrophil elastase in acute lung injury. *Eur J Pharmacol*. 2002; 451: 1–10. 10.1016/s0014-2999(02)02182-9 12223222

[46] Doring G. The role of neutrophil elastase in chronic inflammation. *Am J Respir Crit Care Med*. 1994; 150: S114–7. 10.1164/ajrccm/150.6_Pt_2.S114 7952645

[47] Hammond ME, Lapointe GR, Feucht PH, Hilt S, Gallegos CA, Gordon CA, et al IL-8 induces neutrophil chemotaxis predominantly via type I IL-8 receptors. *J Immunol*. 1995; 155: 1428–33. 7636208

[48] Sahoo M, del Barrio L, Miller MA, Re F. Neutrophil Elastase Causes Tissue Damage That Decreases Host Tolerance to Lung Infection with Burkholderia Species. *PLoS Pathog*. 2014; 10: e1004327 10.1371/journal.ppat.1004327 25166912 PMC4148436

[49] Bardoel Bart W, Kenny Elaine F, Sollberger G, Zychlinsky A. The Balancing Act of Neutrophils. *Cell Host Microbe*. 2014; 15: 526–36. 10.1016/j.chom.2014.04.011 24832448

[50] Middleton EA, Rondina MT, Schwertz H, Zimmerman GA. Amicus or Adversary Revisited: Platelets in Acute Lung Injury and Acute Respiratory Distress Syndrome. *Am J Respir Cell Mol Biol*. 2018; 59: 18–35. 10.1165/rcmb.2017-0420TR 29553813 PMC6039872

[51] Lee KH, Hui KP, Tan WC. Thrombocytopenia in sepsis: a predictor of mortality in the intensive care unit. *Singapore Med J*. 1993; 34: 245–6. 8266183

[52] Wuescher LM, Takashima A, Worth RG. A novel conditional platelet depletion mouse model reveals the importance of platelets in protection against Staphylococcus aureus bacteremia. *J Thromb Haemost*. 2015; 13: 303–13. 10.1111/jth.12795 25418277 PMC4320667

[53] de Stoppelaar SF, van 't Veer C, Claushuis TA, Albersen BJ, Roelofs JJ, van der Poll T. Thrombocytopenia impairs host defense in gram-negative pneumonia-derived sepsis in mice. *Blood*. 2014; 124: 3781–90. 10.1182/blood-2014-05-573915 25301709 PMC4263985

[54] Hechler B, Zimmermann C, Rabouel Y, Magnenat S, Burban M, Boisramé-Helms J, et al A Potential Protective Role of Platelets during Septic Shock Does Not Depend on Their Purinergic Receptors. *Blood*. 2016; 128: 2537–.

[55] Martinod K, Wagner DD. Thrombosis: tangled up in NETs. *Blood*. 2014; 123: 2768–76. 10.1182/blood-2013-10-463646 24366358 PMC4007606

[56] Schauer C, Janko C, Munoz LE, Zhao Y, Kienhofer D, Frey B, et al Aggregated neutrophil extracellular traps limit inflammation by degrading cytokines and chemokines. *Nat Med*. 2014; 20: 511–7. 10.1038/nm.3547 24784231

[57] Looney MR, Nguyen JX, Hu Y, Van Ziffle JA, Lowell CA, Matthay MA. Platelet depletion and aspirin treatment protect mice in a two-event model of transfusion-related acute lung injury. *The Journal of Clinical Investigation*. 2009; 119: 3450–61. 10.1172/JCI38432 19809160 PMC2769181

[58] Luo S, Wang Y, An Q, Chen H, Zhao J, Zhang J, et al Platelets protect lung from injury induced by systemic inflammatory response. *Sci Rep*. 2017; 7: 42080 10.1038/srep42080 28155889 PMC5290476

[59] Abdulnour RE, Dalli J, Colby JK, Krishnamoorthy N, Timmons JY, Tan SH, et al Maresin 1 biosynthesis during platelet-neutrophil interactions is organ-protective. *Proc Natl Acad Sci U S A*. 2014; 111: 16526–31. 10.1073/pnas.1407123111 25369934 PMC4246348

[60] Frangogiannis NG. The inflammatory response in myocardial injury, repair, and remodelling. *Nat Rev Cardiol*. 2014; 11: 255–65. 10.1038/nrcardio.2014.28 24663091 PMC4407144

[61] Lefer AM, Campbell B, Scalia R, Lefer DJ. Synergism Between Platelets and Neutrophils in Provoking Cardiac Dysfunction After Ischemia and Reperfusion. *Role of Selectins*. 1998; 98: 1322–8. 10.1161/01.cir.98.13.1322 9751682

[62] Hargrave B, Li F. Nanosecond pulse electric field activation of platelet-rich plasma reduces myocardial infarct size and improves left ventricular mechanical function in the rabbit heart. *The journal of extra-corporeal technology*. 2012; 44: 198–204. 23441560 PMC4557561

[63] Milioli M, Ibanez-Vea M, Sidoli S, Palmisano G, Careri M, Larsen MR. Quantitative proteomics analysis of platelet-derived microparticles reveals distinct protein signatures when stimulated by different physiological agonists. *J Proteomics*. 2015; 121: 56–66. 10.1016/j.jprot.2015.03.013 25835965

[64] Vélez P, Izquierdo I, Rosa I, García Á. A 2D-DIGE-based proteomic analysis reveals differences in the platelet releasate composition when comparing thrombin and collagen stimulations. *Sci Rep*. 2015; 5: 8198 10.1038/srep08198 25645904 PMC4316189

[65] Chatterjee M, Huang Z, Zhang W, Jiang L, Hultenby K, Zhu L, et al Distinct platelet packaging, release, and surface expression of proangiogenic and antiangiogenic factors on different platelet stimuli. *Blood*. 2011; 117: 3907–11. 10.1182/blood-2010-12-327007 21330475

[66] Pokrovskaya ID, Aronova MA, Kamykowski JA, Prince AA, Hoyne JD, Calco GN, et al STEM tomography reveals that the canalicular system and alpha-granules remain separate compartments during early secretion stages in blood platelets. *J Thromb Haemost*. 2016; 14: 572–84. 10.1111/jth.13225 26663480 PMC4829117

