# Supplementary figures and images for: Platelets modulate multiple markers of neutrophil function in response to in vitro Toll-like receptor stimulation
Source: PLoS One. 2019 Oct 3;14(10):e0223444. doi: 10.1371/journal.pone.0223444 (PMC6776355; doi:10.1371/journal.pone.0223444)

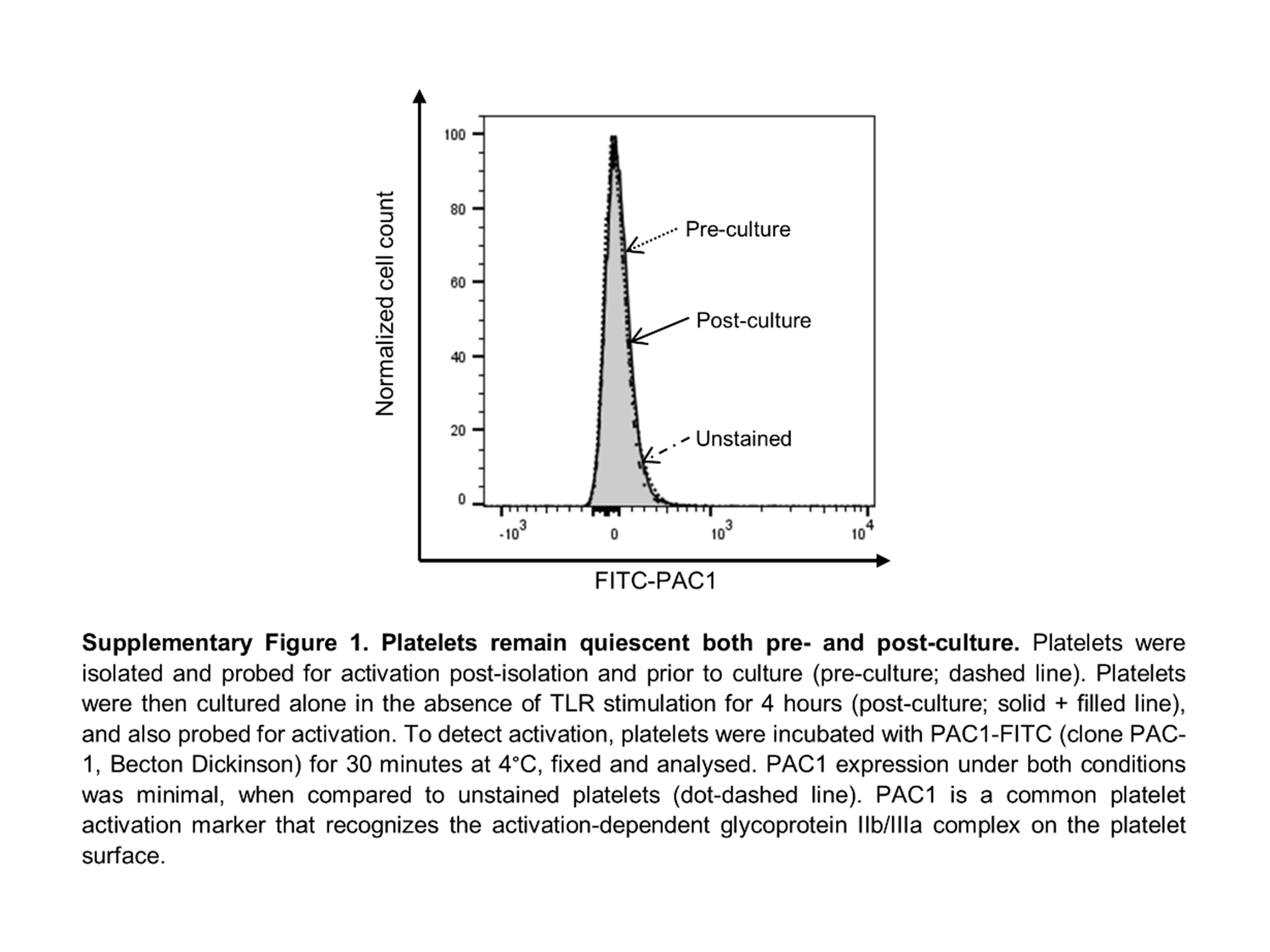

Supplement: S1 Fig — Platelets were isolated and probed for activation post-isolation and prior to culture (pre-culture; dashed line). Platelets were then cultured alone in the absence of TLR stimulation for 4 hours (post-culture; solid + filled line), and also probed for activation. To detect activation, platelets were incubated with PAC1-FITC (clone PAC-1, Becton Dickinson) for 30 minutes at 4°C, fixed and analysed. PAC1 expression under both conditions was minimal, when compared to unstained platelets (dot-dashed line). PAC1 is a common platelet activation marker that recognizes the activation-dependent glycoprotein IIb/IIIa complex on the platelet surface. (TIF) [file pone.0223444.s001.tif]

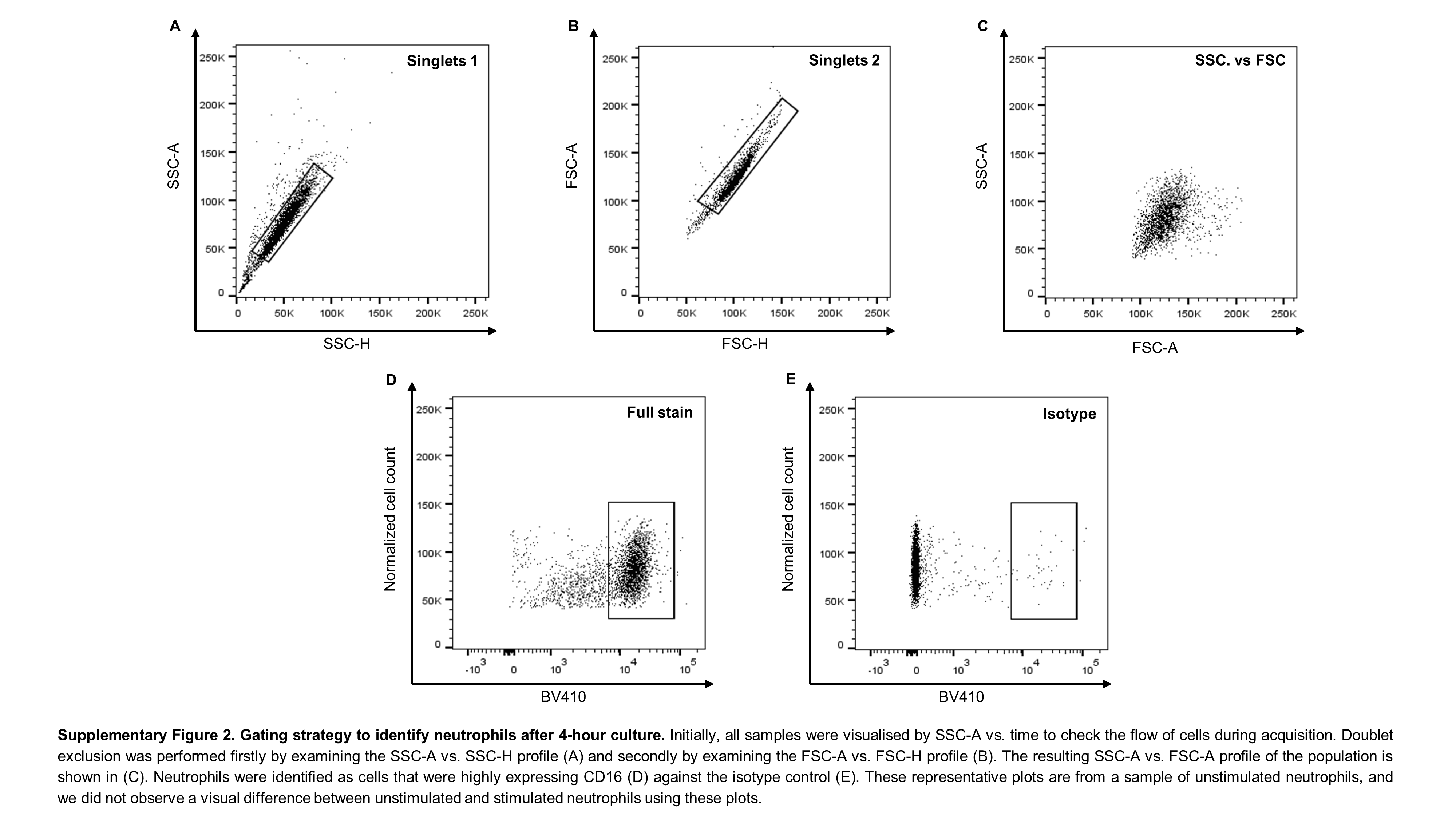

Supplement: S2 Fig — Initially, all samples were visualised by SSC-A vs. time to check the flow of cells during acquisition. Doublet exclusion was performed firstly by examining the SSC-A vs. SSC-H profile (A) and secondly by examining the FSC-A vs. FSC-H profile (B). The resulting SSC-A vs. FSC-A profile of the population is shown in (C). Neutrophils were identified as cells that were highly expressing CD16 (D) against the isotype control (E). These representative plots are from a sample of unstimulated neutrophils, and we did not observe a visual difference between unstimulated and stimulated neutrophils using these plots. (TIF) [file pone.0223444.s002.tif]

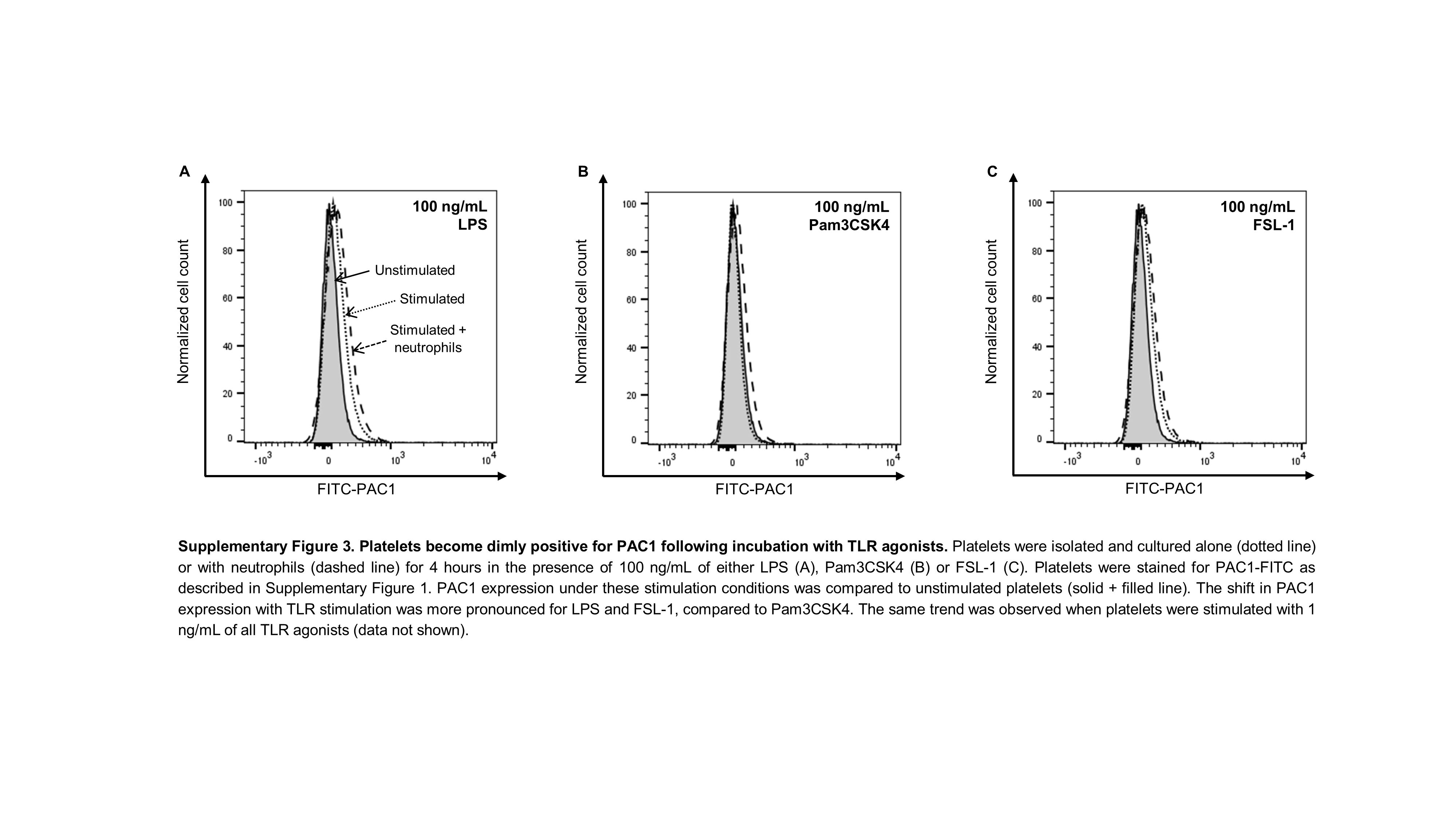

Supplement: S3 Fig — Platelets were isolated and cultured alone (dotted line) or with neutrophils (dashed line) for 4 hours in the presence of 100 ng/mL of either LPS (A), Pam3CSK4 (B) or FSL-1 (C). Platelets were stained for PAC1-FITC as described in S2 Fig. PAC1 expression under these stimulation conditions was compared to unstimulated platelets (solid + filled line). The shift in PAC1 expression with TLR stimulation was more pronounced for LPS and FSL-1, compared to Pam3CSK4. The same trend was observed when platelets were stimulated with 1 ng/mL of all TLR agonists (data not shown). (TIF) [file pone.0223444.s003.tif]

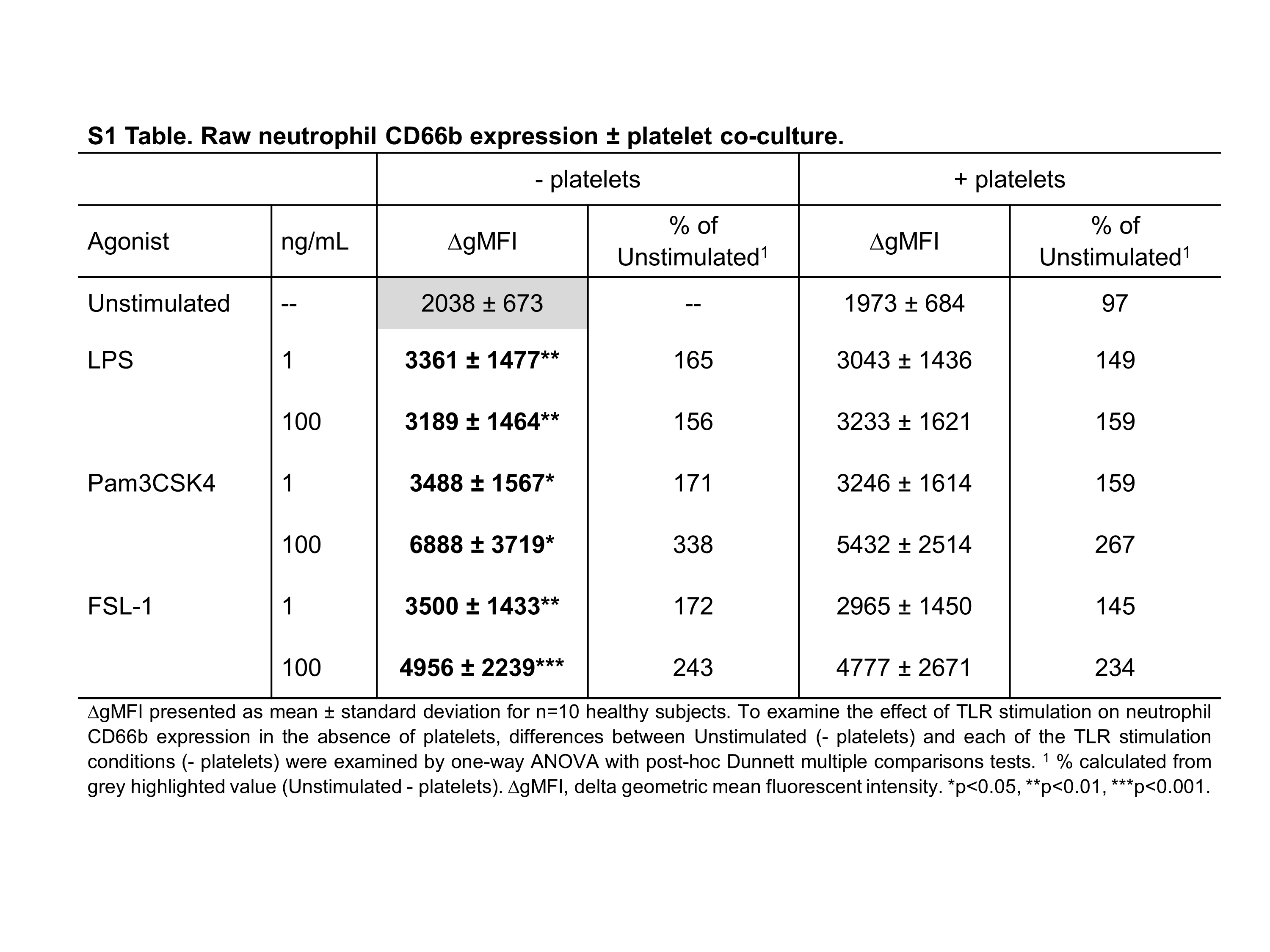

Supplement: S1 Table — (TIF) [file pone.0223444.s004.tif]

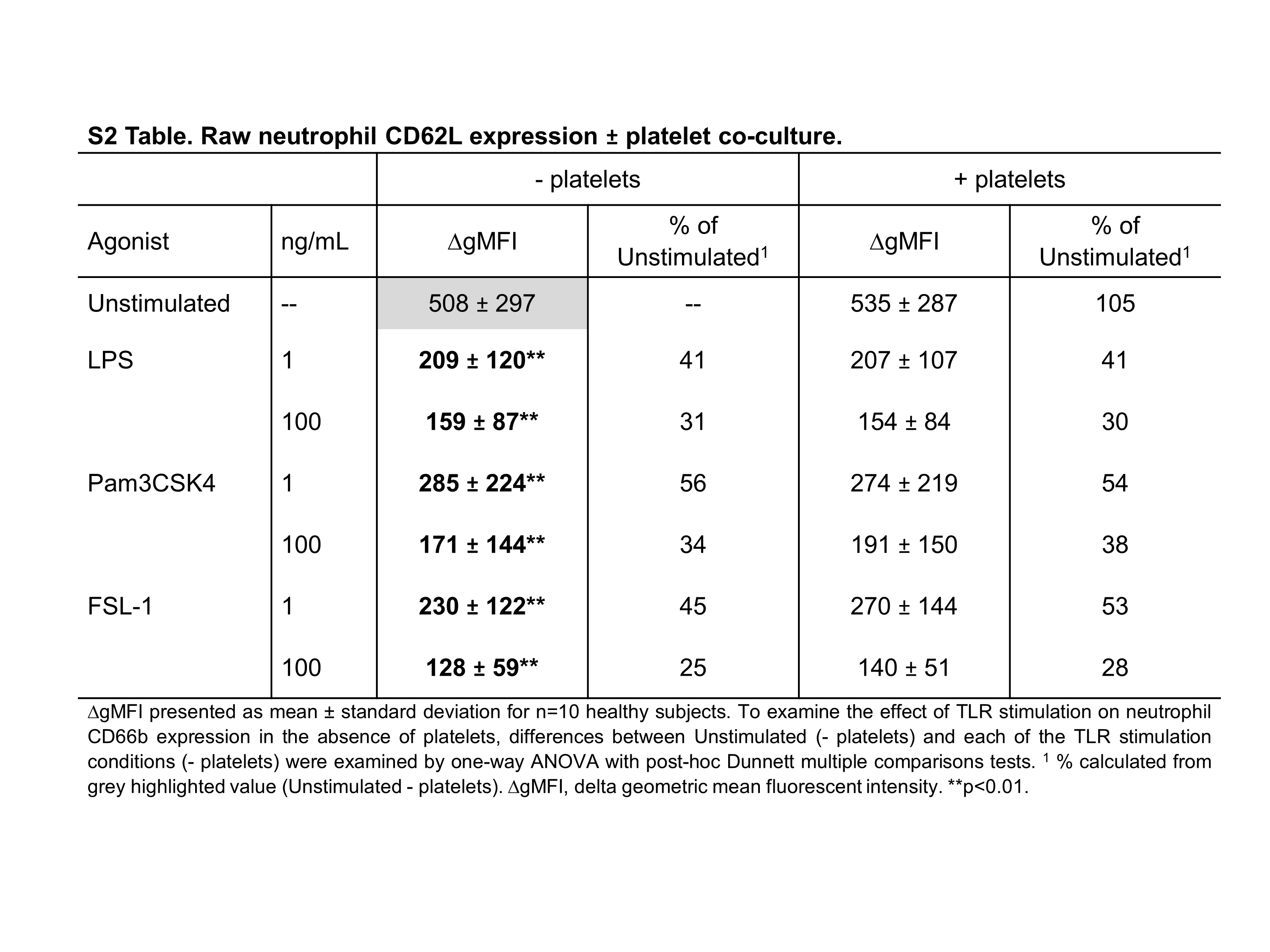

Supplement: S2 Table — (TIF) [file pone.0223444.s005.tif]

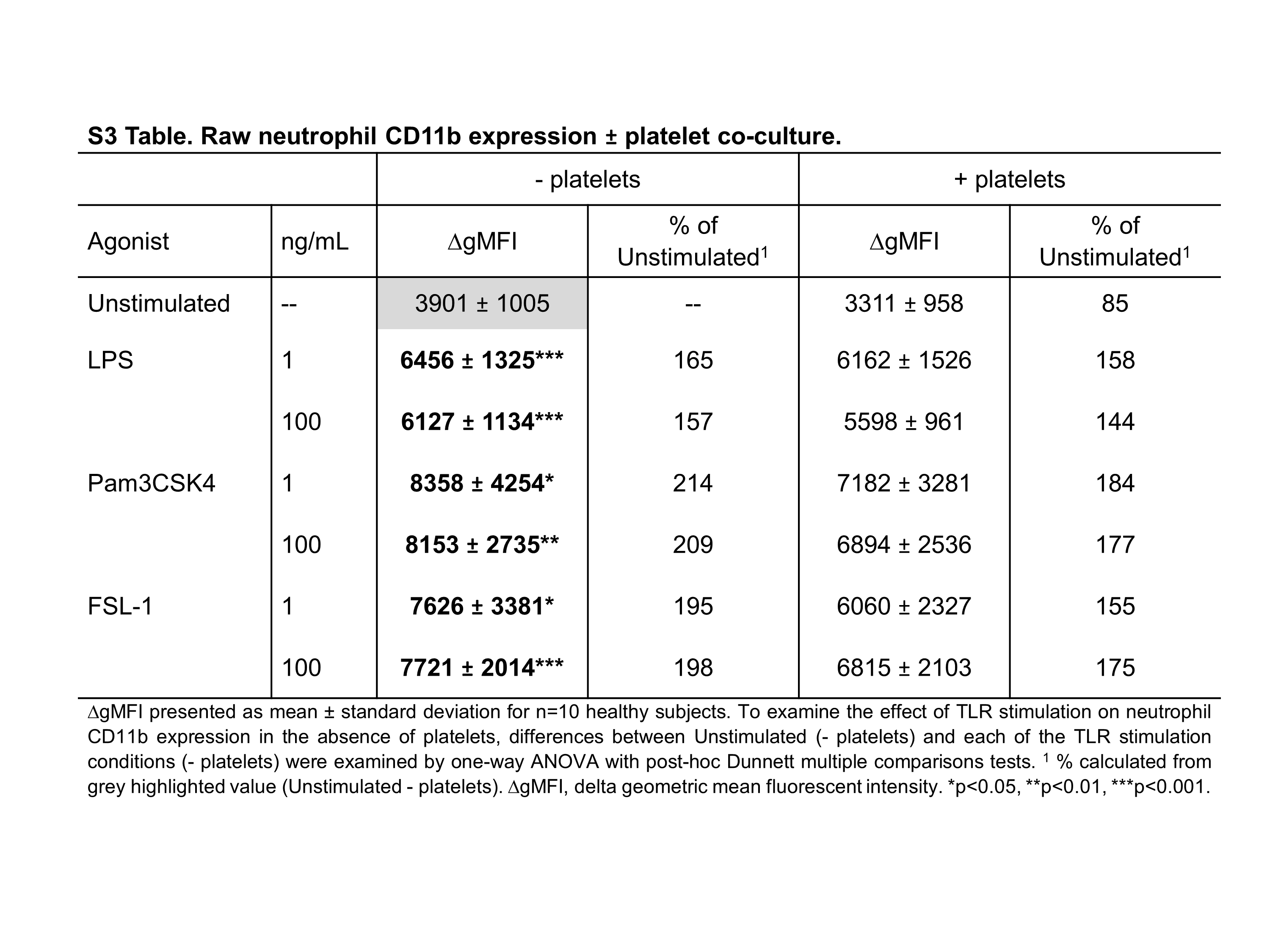

Supplement: S3 Table — (TIF) [file pone.0223444.s006.tif]

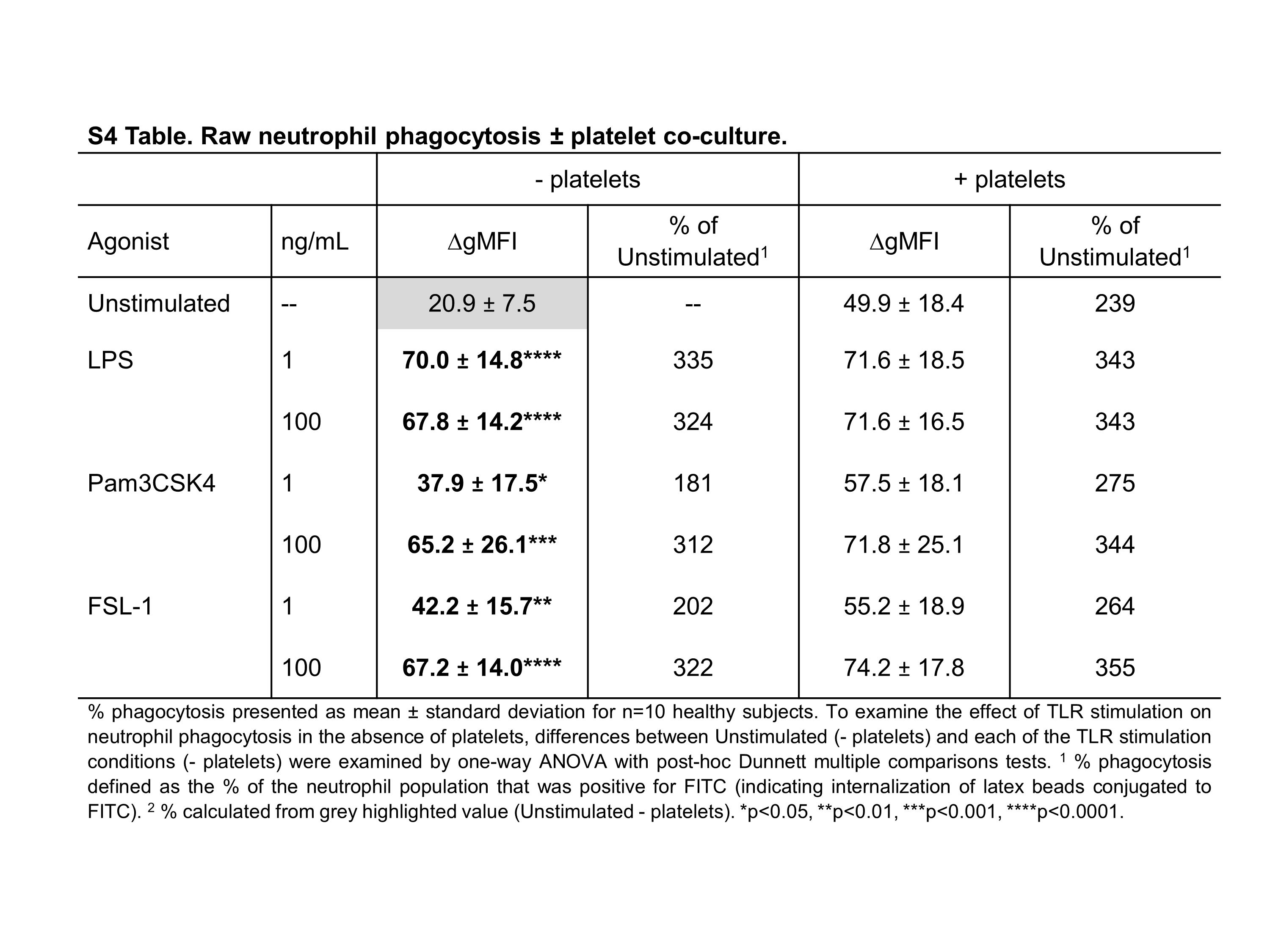

Supplement: S4 Table — (TIF) [file pone.0223444.s007.tif]

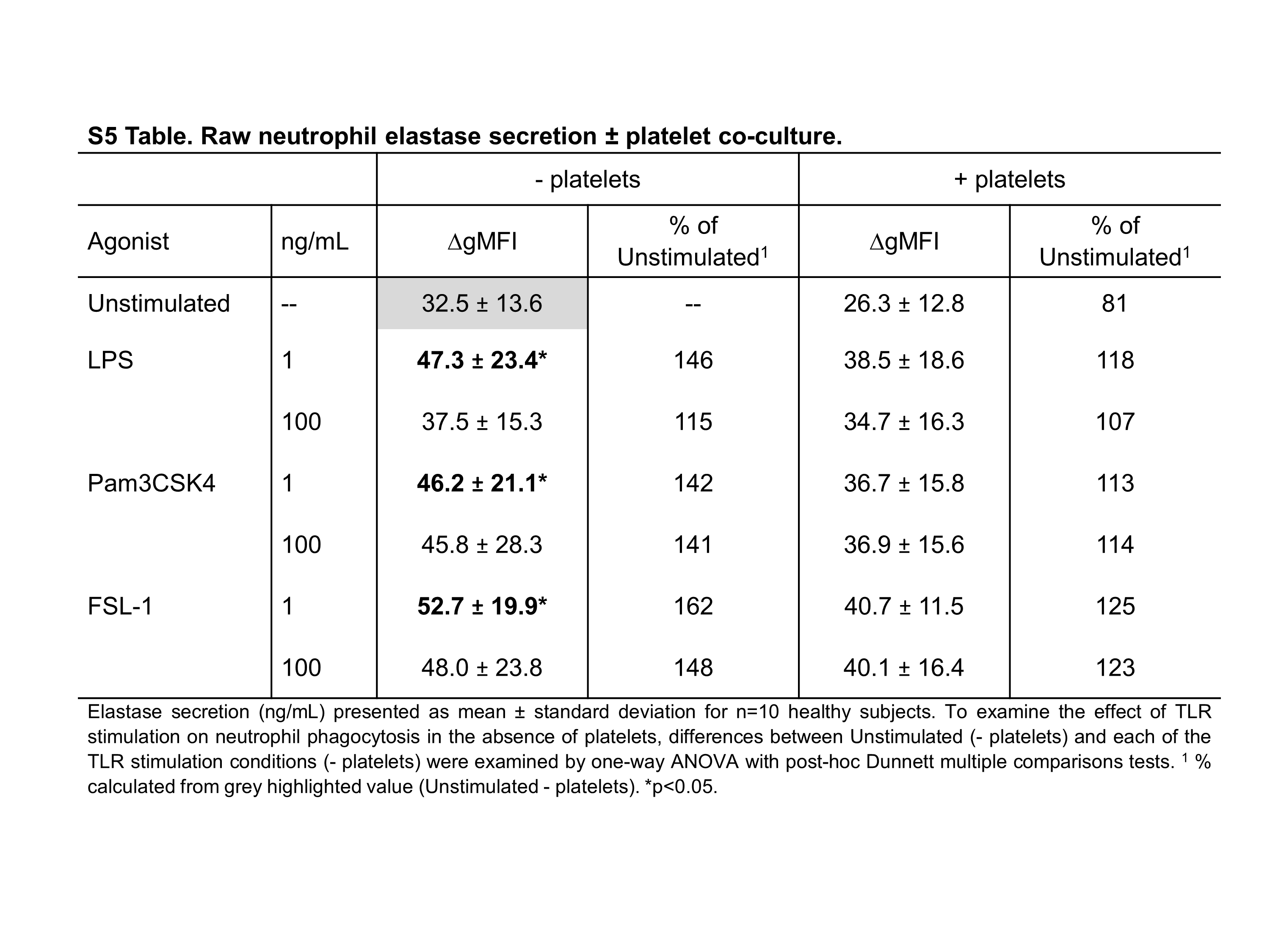

Supplement: S5 Table — (TIF) [file pone.0223444.s008.tif]

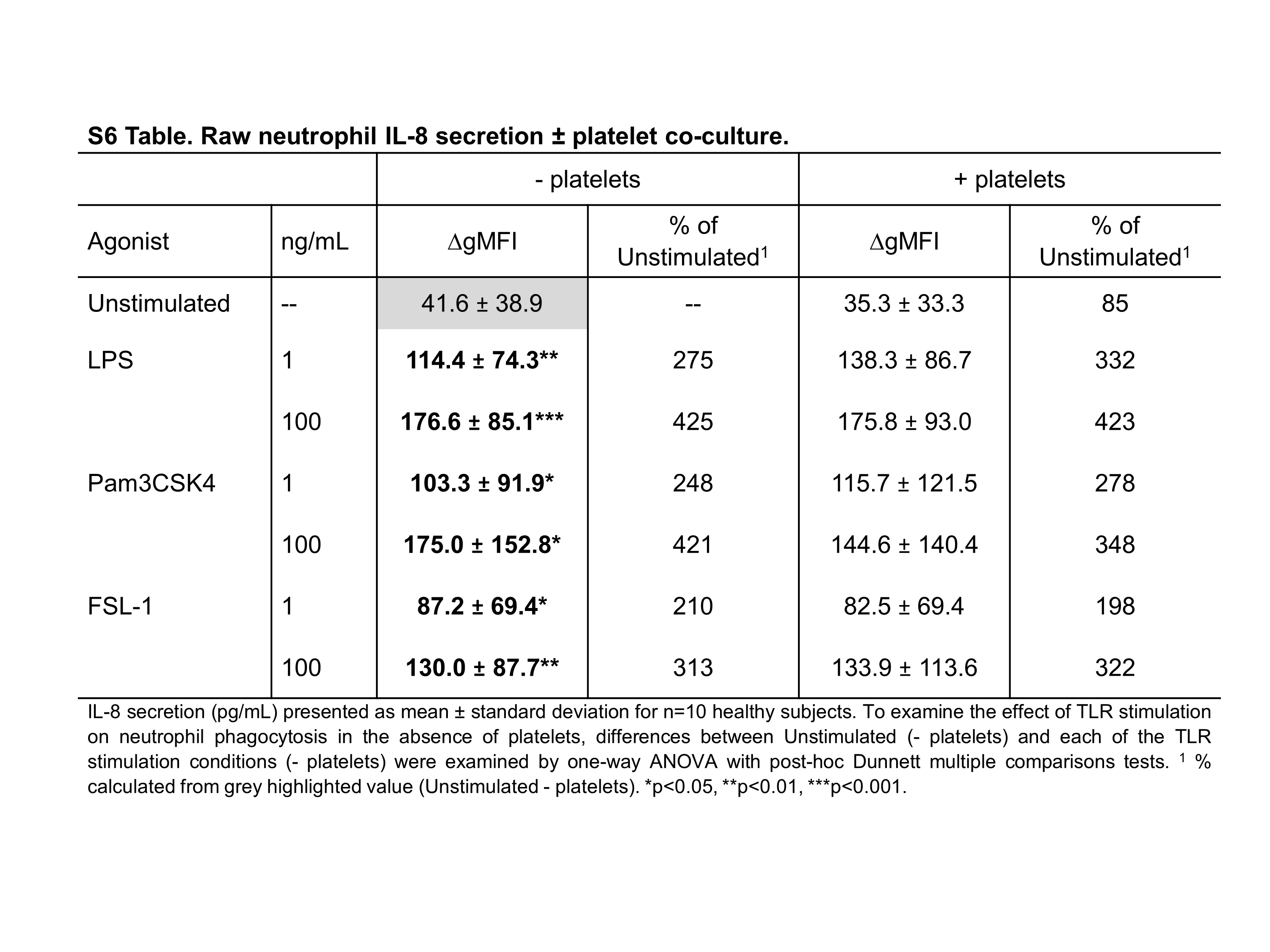

Supplement: S6 Table — (TIF) [file pone.0223444.s009.tif]
